# Supplementary material for: Investigation to identify individual socioeconomic and health determinants of suicidal ideation using responses to a cross-sectional, community-based public health survey
Source: BMJ Open. 2021 Feb 4;11(2):e035252. doi: 10.1136/bmjopen-2019-035252 (PMC7868260; doi:10.1136/bmjopen-2019-035252)
Supplement: Supplementary data [file bmjopen-2019-035252supp001.pdf]

Supplementary File

Study measures - description

Suicidal ideation (SI)

Suicidal ideation was assessed utilising item 9 of the Patient and Health Questionnaire (PHQ-9).[27] Participants indicated how often over the last two weeks they had been bothered by "thoughts that you would be better off dead, or of hurting yourself in some way". Response options ranged from 1 = *not at all* to 4 = *every day*.

Socio-economic demographics

Participants completed demographic measures, which included age, sex, ethnicity, sexuality, and relationship status. Measures of socioeconomic conditions included education level, employment status, change in financial circumstances over the past year, and housing quality.

Health

Physical health was assessed with the four physical health dimensions of the *EuroQuol five-dimension scale* (EQ-5D).[31] Health conditions were assessed with a single item from the *Adult Psychiatric Morbidity Survey: Survey of Mental Health and Wellbeing, England*[33] that allowed participants to indicate if they suffered from any of 22 named conditions, with the option to list an unnamed condition. Using this item, physical and mental health comorbidity was calculated and categorised into no conditions, mental health condition/s only, physical health condition/s only, and physical and mental health condition/s. Participants also indicated the extent to which medication side effects bothered them. Mental health was assessed using a series of validated instruments. Specifically, depression was measured using the nine-item PHQ-9[27] with item 9 (suicidal ideation) excluded as it was used as the dependent variable; anxiety was measured with the seven-item *Generalised Anxiety Disorder* scale (GAD-7),[36] paranoia was measured using the

persecution subscale of the *Persecution and Deservedness Scale* for symptoms of paranoia (PaDS-5),[37] and wellbeing was assessed with the *Warwick-Edinburgh Mental Wellbeing Scale* (WEMWBS).[38] Measures of psychological variables known to be associated with poor mental health were also obtained, including the *Single-Item Self Esteem Scale* (SISES),[39] along with abbreviated scales for empathy (Interpersonal Reactivity Index),[40] hopelessness (*Brief-H-Pos;* reverse scored)[41] and locus of control (Multidimensional Locus of Control Scales).[42]

Lifestyle factors

Alcohol consumption was measured using the number of units of alcohol participants reported consuming in the previous seven days. Participants were categorised in accordance with recommended drinking guidelines[66] as: abstaining, at or below recommended, above recommended, or more than double recommended levels. Past and present smoking behaviour was assessed with a single item adapted from the NHS Merseyside Lifestyle Survey.[35]

Social connectedness/capital

Participants indicated the number of hours they spent caring for a friend or family member. Social support, socialising, and neighbourhood belonging were assessed with single items from the *Community Life Survey*.[32]

Supplementary File Table 1. Coding and source of study measures

Variable	Source	Description/Example	Original scoring	Recoding for analysis
Age	Office for National Statistics	N/A	1 = Under 16 years<br>2 = 16-17 years<br>3 = 18-24 years<br>4 = 25-34 years<br>5 = 35-44 years<br>6 = 45-54 years<br>7 = 55-64 years<br>8 = 65-74 years<br>9 = 75+ years	1 = 18-24 years<br>2 = 25-44 years<br>3 = 45-64 years<br>4 = 65+ years

Sex	Office for National Statistics	N/A	1 = Male<br>2 = Female<br>3 = Other	1 = Male<br>2 = Female
Sexuality	Office for National Statistics	N/A	1 = Heterosexual or straight<br>2 = Gay or Lesbian<br>3 = Bisexual<br>4 = Other	0 = heterosexual<br>1 = LGBTQ
Ethnicity	Office for National Statistics	N/A	1 = English / Welsh / Scottish / Northern Irish / British<br>2 = Irish<br>3 = Gypsy or Irish Traveller<br>4 = Any other White background, *please specify*<br>5 = White and Black Caribbean	0 = White<br>1 = BME

			6 = White and Black African<br><br>7 = White and Asian<br><br>8 = Any other Mixed / Multiple ethnic background, *please specify*<br><br>9 = Indian<br><br>10 = Pakistani<br><br>11 = Bangladeshi<br><br>12 = Chinese<br><br>13 = Any other Asian background, *please specify*<br><br>14 = African<br><br>15 = Caribbean<br><br>16 = Any other Black / African / Caribbean background, *please specify*	

			17 = Arab<br><br>95 = Any other group, please specify	
Relationship status	Office for National Statistics	N/A	1 = Single/never married or in a same-sex civil partnership<br><br>2 = Married<br><br>3 = Separated, but still legally married<br><br>4 = Divorced<br><br>5 = Widowed<br><br>6 = Same-sex civil partnership<br><br>7 = Separated, but still in a same-sex civil partnership<br><br>8 = Formerly in a same-sex civil partnership that is now dissolved	0 = Partnered<br><br>1 = Single

			9 = Surviving partner from a same-sex civil partnership<br>10 = prefer not to say	
Non-employment	Office for National Statistics	N/A	1 = Going to school or college full time (including on vacation)<br>2 = In paid employment or self employed (or temporarily away)<br>3 = On a Government scheme for employment training<br>4 = Doing unpaid work for a business that you own, or that a relative owns<br>5 = Waiting to take up paid work already obtained<br>6 = Looking for paid work or a Government training scheme	0 = employed<br>1 = not employed

			7 = Intending to look for work but prevented by temporary sickness or injury<br>8 = Permanently unable to work because of long-term sickness or disability<br>9 = Retired from paid work<br>10 = Looking after the home or family<br>95 = Doing something else, specify	
Education	Office for National Statistics	Do you have any educational qualifications for which you received a certificate?<br><br>Do you have any professional, vocational or other work-related	1 = Yes, 2 = No<br><br>1 = Yes, 2 = No	1 = No qualifications<br>2 = Professional or vocational qualification<br>3 = Degree or higher

		qualifications for which you received a certificate?<br><br>What is your highest qualification?	1 = At degree level or above, 2 = Another kind of qualification	
Caring responsibilities	UK Census	Do you look after, or give any help or support to family members, friends, neighbours or others because of either a long term physical or mental ill-health / disability or problems related to old age? Do not count	1 = No<br><br>2 = Yes, 1-19 hours/week<br><br>3 = Yes, 20-49 hours/week<br><br>4 = Yes, 50+ hours/week	N/A

		anything you do as part of your paid employment.		
Housing quality	English Housing Survey	During the winter months, does condensation form on the windows or walls of any room in your home apart from the bathrooms or toilets?<br><br>During the winter months, are there patches of mould or fungus in any room in your home, apart from bathrooms or toilets?	1 = Yes, 2 = No, 3 = Spontaneous: don't know<br><br>1 = Yes, 2 = No, 3 = Spontaneous: don't know	0 = No problems<br>1 = One problem<br>2 = Two problems<br>3 = Problems with all three issues

		During the cold winter weather, can you normally keep comfortably warm in your living room?	1 = Yes, 2 = No, 3 = Spontaneous: don't know	
Financial Situation	Wealth and Assets Survey	Would you say your household is better off or worse off financially than you were a year ago?	1 = Better off<br>2 = About the same<br>3 = Worse off<br>4 = Don't know	1 = Better off<br>2 = About the same<br>3 = Worse off
Physical Health status	EuroQual Five Dimensional	Standardised instrument that assesses problems with mobility, self-care, engagement in usual activities, and pain.	1 = No problems<br>2 = Some Problems/Moderate problems<br>3 = Extreme problems/Unable	0 = No problems<br>1 = Some/Severe problems

	Scale (EQ-5D)			
Physical health conditions	Psychiatric Morbidity Survey	Have you ever had any of [these health conditions] over the past 12 months?	1 = Cancer<br>2 = Diabetes<br>3 = Epilepsy/fits<br>4 = Migraine or other frequent headaches<br>5 = Dementia or Alzheimer's disease<br>6 = Any mental health issue<br>7 = Cataracts / eyesight problems (even if corrected with glasses or contacts)<br>8 = Ear/hearing problems (even if corrected with a hearing aid)<br>9 = Stroke	For each condition:<br>0 = condition absent<br>1 = condition present<br>For physical and mental health comorbidity:<br>0 = No conditions

			10 = Heart attack/angina<br>11 = High blood pressure<br>12 = Bronchitis/emphysema<br>13 = Asthma<br>14 = Allergies<br>15 = Stomach ulcer or other digestive problems<br>16 = Liver problems<br>17 = Bowel/colon problems<br>18 = Bladder problems/incontinences<br>19 = Arthritis<br>20 = Bone, back joint or muscle problems<br>21 = Gout	1 = Mental health condition(s) only<br>2 = Physical health conditions(s) only<br>3 = Physical and mental health condition

			22 = Skin problems<br><br>95 = Any other condition	
Side effects	Health Survey for England (HSE)	Do any of your medications cause side effects or bother you in any way?<br><br>If yes, how much does it bothers you?	1 = Yes<br><br>2 = No<br><br>1 = A lot<br><br>2 = Somewhat<br><br>3 = A little<br><br>4 = Never	0 = No medication<br><br>1 = Never bothers<br><br>2 = Bothers a little<br><br>3 = Bothers somewhat<br><br>4 = Bothers a lot
Depression	Patient Health	Assesses how often participants had been bothered by pro problems such as "Feeling	1 = Not at all<br><br>2 = Several days	N/A

	Questionnaire (PHQ-9)	down, depressed, or hopeless" over the past two weeks.	3 = More than half the days<br>4 = Nearly every day	
Anxiety	Generalised Anxiety Disorder Questionnaire (GAD-7)	Assesses frequency of events such as "Being so restless that it is hard to sit still" over the past two weeks.	1 = Not at all<br>2 = Several days<br>3 = More than half the days<br>4 = Nearly every day	N/A
Paranoia	Five-item Persecution and Deservedness Scale (PaDS-5)	Assesses the extent to which people are suspicious of others' intentions. Participants rate their level of agreement with statements such as "I'm often suspicious of other people's intentions towards me."	1 = Strongly disagree<br>2 = Disagree<br>3 = Neither agree or disagree<br>4 = Agree<br>5 = Strongly agree	N/A

Wellbeing	The Warwick-Edinburgh Mental Well-being Scale (WEMWBS)	Abbreviated 7-item scale. Assesses general mood and wellbeing over the previous 2 weeks. Example statements include "I've been feeling relaxed" and "I've been thinking clearly".	1 = None of the time<br>2 = Rarely<br>3 = Some of the time<br>4 = Often<br>5 = All of the time	N/A
Self-esteem	Single-item Self-esteem Scale (SISE)	Validated scale assessing general levels of self-esteem. Participants rate their level of agreement on a 7-point scale with the statement "I have high self-esteem".	1 = Not very true of me<br>7 = Very true of me	

Empathy	Interpersonal Reactivity Index (IRI)	Abbreviated five-item version. Participants rate their level of agreement with statements such as "I am good at predicting how someone will feel".	1 = Strongly disagree<br>2 = Disagree<br>3 = Neither agree or disagree<br>4 = Agree<br>5 = Strongly agree	
Locus of control	Levenson Locus of Control Scale	Abbreviated nine-item version. Participants rated their level of agreement with statements such as "My life is determined by my own actions".	1 = Strongly disagree<br>2 = Disagree<br>3 = Neither agree or disagree<br>4 = Agree<br>5 = Strongly agree	N/A
Hopelessness	Brief-H-Pos	Participants rated their level of agreement with two statements: "The future seems to me to be	1 = Strongly disagree<br>2 = Disagree	1 = Strongly agree<br>2 = Agree

		hopeful and I believe that things are changing for the better" and "I feel that it is possible to reach the goals I would like to strive for"	3 = Neither agree or disagree<br>4 = Agree<br>5 = Strongly agree	3 = Neither agree or disagree<br>4 = Disagree<br>5 = Strongly disagree
Alcohol	Merseyside Lifestyle Survey	Participants indicated if they ever drank alcohol and if so how many of the following drinks they had consumed over the past seven days: pints of beer (low, normal and strong), pints of cider, bottles of alcopops, glasses of spirits, glasses of wine (small and large), glasses of fortified wine. These numbers were converted to	1 = none, moderate (<14 units/week),<br>2 = heavy (14-28 units/week),<br>3 = very heavy (>28 units/week).	N/A

		alcoholic units and then people were categorized into four levels of alcohol consumption based on the recommended usage of less than 14 units per week (Department of Health, 2016)		
Smoking status	Merseyside Lifestyle Survey	N/A	1 = Never smoked<br>2 = Past occasional smoker<br>3 = Past daily smoker<br>4 = Current occasional smoker<br>5 = Current daily smoker	N/A
Social capital	Community Life Survey	Assesses the extent to which participants agree they receive	1 = Definitely agree	0 = Disagree

		practical support ("If I needed help, there are people who would be there for me") and *social contact* ("If I wanted company or to socialise, there are people I can call on")	2 = Tend to agree<br>3 = Tend to disagree<br>4 = Definitely disagree<br>5 = Don't know	1 = Agree
Neighbourhood belonging	Community Life Survey	Participants were asked "how strongly you feel you belong to your immediate neighbourhood".	1 = Very strongly<br>2 = Fairly strongly<br>3 = Not very strongly<br>4 = Not at all strongly<br>5 = Don't know	0 = Not very/at all strongly<br>1 = Fairly/very strongly

Supplementary File Table 2. *Descriptive statistics for all variables. Discrepancies between total values and crosstabulated values due to non-response on the suicidal ideation variable*

	N (%)	*M* (*SD*)	*N*(%) *No suicidal ideation*	*N*(% *Suicidal ideation*
**Suicidal ideation in previous 2 weeks**				
Suicidal ideation	454 (10.6)	-	-	-
No suicidal ideation	3833 (89.4)	-	-	-
**Mental health symptoms**				
Depression (PHQ-9)	-	4.65(5.77)	-	-
Anxiety (GAD-7)	-	9.00 (4.33)	-	-
Paranoia (PaDS-5)	-	9.68 (4.38)	-	-
**Age**				
18-24 years	421 (9.8)	-	368(87.6)	52 (12.4)
25-44 years	1438 (33.3)	-	1247 (87.3)	181 (12.7)
45-64 years	1329 (30.8)	-	1162 (87.9)	160 (12.1)
65+ years	1129 (26.2)	-	1055 (94.6)	60 (5.4)
**Gender**				
Female	2465 (57.1)	-	2205 (90.2)	249 (9.8)
Male	1854 (42.9)	-	1628 (88.4)	214 (11.6)
**Ethnicity**				
Black and Minority Ethnic	455 (10.6)	-	414 (91.6)	38 (8.4)
White	3855 (89.4)	-	3411 (89.2)	415 (10.9)
**Sexuality**				
LGBTQ	65 (1.5)	-	50 (76.9)	15 (23.08)

Not LGBTQ	4246 (98.5)	-	3775 (89.6)	439 (10.4)
**Relationship status**				
Not married/civil partnership	2559 (49.4)	-	2209 (87.0)	330 (13.0)
Married/civil partnership	1747 (40.6)	-	1613 (92.9)	123 (7.1)
**Education**				
No qualifications	1718 (39.9)	-	1498 (88.0)	205 (12.0)
Professional/vocational certificate	1931 (44.8)	-	1727 (90.0)	192 (10.0)
Degree or higher	659 (15.3)	-	599 (91.3)	57 (8.7)
**Employment status**				
Employed	1745 (40.4)	-	1608 (92.5)	130 (7.5)
Not employed	2570 (59.6)	-	2233 (87.3)	323 (12.7)
**Housing quality (0-1 scale)**	-	.32 (.47)	-	-
-				
**Financial position compared with 12 months ago**				
Worse	679 (15.7)	-	549 (81.7)	123 (18.3)
Same	3090 (72.2)	-	2781 (90.6)	289 (9.4)
Better	510 (11.8)	-	471 (92.7)	37 (7.3)
**Caring responsibilities**				
None	3716 (86.0)	-	3298 (89.5)	389 (10.5)
1-19 hours/week	275 (6.4)	-	250 (91.6)	23 (8.4)
20-49 hours/week	100 (2.3)	-	86 (86.0)	14 (14.0)
50+ hours/week	228 (5.3)	-	199 (87.7)	28 (12.3)
**Health problems (EQ-5D; 0 – 1 scale)**				

Mobility	-	.24 (.43)	-	-
Self-care	-	.10 (.29)	-	-
Usual activities	-	.22 (.42)	-	-
Pain	-	.36 (.48)	-	-
**Health conditions**				
Cancer	120 (2.8)	-	99 (83.4)	20 (16.8)
Diabetes	341 (7.9)	-	299 (87.7)	42 (12.3)
Epilepsy	80 (1.9)	-	52 (65.8)	27 (34.2)
Migraine	348 (8.1)	-	270 (78.5)	74 (21.5)
Dementia	21 (0.5)	-	17 (81.0)	4 (19.0)
Eye	418 (9.7)	-	351 (85.2)	61 (14.8)
Ear	247 (5.7)	-	206 (85.1)	36 (14.9)
Stroke	97 (2.3)	-	76 (78.4)	21 (21.6)
Heart	280 (6.5)	-	240 (87.0)	36 (13.0)
Blood pressure	711 (16.5)	-	618 (88.0)	84 (12.0)
Bronchitis/Emphysema	113 (2.6)	-	91 (81.3)	21 (18.7)
Asthma	418 (9.7)	-	356 (85.2)	62 (14.8)
Allergies	185 (4.3)	-	158 (86.3)	25 (13.7)
Stomach/digestive	224 (5.2)	-	185 (83.0)	38 (17.0)
Liver	73 (1.7)	-	50 (70.4)	21 (29.6)
Bowel/colon	173 (4.0)	-	133 (77.8)	38 (22.2)
Bladder	136 (3.2)	-	108 (80.0)	27 (20.0)
Arthritis	728 (16.9)	-	634 (88.1)	86 (11.9)
Bone, back, joint, muscle	778 (18.0)	-	667 (86.4)	105 (13.6)
Gout	51 (1.2)	-	47 (92.2)	4 (7.8)
Skin	243 (5.6)	-	211 (87.6)	30 (12.4)
Other	267 (6.2)	-	235 (88.0)	32 (12.0)
**Side effects**				

No medication	2015 (46.8)	-	1842 (92.0)	160 (8.0)
Never bothers	1945 (45.2)	-	1732 (89.7)	198 (10.3)
Bothers a little	79 (1.8)	-	62 (78.5)	17 (21.5)
Bothers somewhat	145 (3.4)	-	106 (74.1)	37 (25.9)
Bothers a lot	125 (2.9)	-	85 (69.1)	38 (30.9)
**Alcohol consumption**				
Never (0 units/week)	1894 (44.0)	-	1674 (89.2)	202 (10.8)
Moderate (1-14 units/week)	1973 (45.8)	-	1761 (89.7)	202 (10.3)
High (14-28 units/week)	290 (6.7)	-	260 (90.0)	29 (10.0)
Very high (>28 units/week)	151 (3.5)	-	130 (87.3)	19 (12.7)
**Smoking status**				
Never	2107 (48.8)	-	1951 (93.3)	141 (6.7)
Past occasional smoking	286 (6.6)	-	257 (89.9)	29 (10.1)
Past daily smoking	671 (15.5)	-	605 (90.8)	61 (9.2)
Current occasional smoking	122 (2.8)	-	97 (79.5)	25 (20.5)
Current daily smoking	1118 (25.9)	-	910 (82.2)	197 (17.8)
**Psychological factors**				
Empathy	-	3.35 (.88)	-	-
Self-esteem	-	4.54 (1.73)	-	-
Hopelessness	-	2.48 (.97)	-	-
Locus of control (power)	-	2.83 (.88)	-	-
Locus of control (chance)	-	2.76 (.74)	-	-
Locus of control (internal)	-	3.65 (.69)	-	-
**Social capital and belonging**				
Practical support	4084 (94.8)	-	3657 (90.2)	397 (9.8)
People to socialise with	4064 (94.4)	-	3642 (90.2)	395 (9.8)

Neighbourhood belonging	3518 (82.1)	-	3176 (90.8)	322 (9.21)

Supplementary File Table 3. *Logistic regression predicting suicidal ideation excluding (Model 1) and including (Model 2) mental health variables.*

	Model 1			*Model 2*	
Predictors	*Adjusted Odds Ratio of suicidal ideation*	*95% CI*		*Adjusted Odds Ratio of suicidal ideation*	*95% CI*
Mental health					
Depression	-	-		*7.24****	*5.22, 10.07*
Anxiety	-	-		*1.56***	*1.13, 2.17*
Paranoia	-	-		*1.36**	*1.72*
Wellbeing	-	-		*.76*	*.56, 1.01*
Demographics					
Age (65+)					
18-24	*5.50****	*2.74, 11.06*		*0.95*	*.38, 2.38*
25-44	*4.50****	*2.48, 8.15*		*1.62*	*.84, 3.15*
45-64	*2.82****	*1.68, 4.73*		*1.1*	*.60, 2.02*
Female	*.88*	*.67, 1.17*		*.81*	*.57, 1.14*
Black & Minority Ethnic (BME)	*1.88**	*1.01, 3.49*		*1.93**	*1.04, 3.62*
LGBTQ	*1.93*	*.77, 4.83*		*2.73**	*1.00, 7.46*
Single/never married or civil partnership	*1.16*	*.87, 1.56*		*1.07*	*.74, 1.56*

Socioeconomic status					
Education (No qualifications)					
Professional, vocational or work Certificate	*.97*	*.71, 1.34*		*.76*	*.52, 1.12*
Degree or higher	*1.06*	*.67, 1.67*		*.70*	*.40, 1.21*
Non-employment	*1.43**	*1.00, 2.03*		*1.06*	*.68, 1.65*
Problems with housing	*1.67****	*1.26, 2.23*		*1.34*	*.95, 1.89*
Financial position (worse)					
Same	*1.68**	*1.02, 2.76*		*2.29***	*1.24, 4.23*
Better	*1.59*	*.91, 2.77*		*1.19*	*.58, 2.42*
Caring responsibilities					
None					
1-19 hours/week	*0.69*	*.38, 1.24*		*.64*	*.32, 1.30*
20-49 hours/week	*1.24*	*.61, 2.53*		*1.00*	*.39, 2.57*
50+ hours/week	*1.23*	*.65, 2.33*		*.68*	*.31, 1.49*
Health problems (EQ-5D)					
Pain	*1.62**	*1.09, 2.40*		*0.98*	*.61, 1.56*
Self-care	*1.38*	*.83, 2.27*		*1.02*	*.50, 2.08*
Usual activities	*1.02*	*.64, 1.62*		*0.64*	*.35, 1.18*
Mobility	*1.00*	*.64, 1.55*		*1.12*	*.65, 1.93*
Health conditions					
Cancer	*1.74*	*.80, 3.77*		*3.90***	*1.40, 10.84*
Diabetes	*1.03*	*.58, 1.83*		*.86*	*.43, 1.75*
Epilepsy	*1.73*	*.98, 3.06*		*1.65*	*.80, 3.39*

Migraine	*1.27*	*.83, 1.94*		*0.73*	*.39, 1.35*
Dementia	*.71*	*.26, 1.98*		*0.29*	*.06, 1.47*
Eye	*.97*	*.64, 1.48*		*1.23*	*.74, 2.06*
Ear	*2.02***	*1.20, 3.41*		*1.24*	*.59, 2.59*
Stroke	*2.01**	*1.06, 3.81*		*1.63*	*.57, 4.68*
Heart	*.80*	*.47, 1.37*		*1.3*	*.68, 2.48*
Blood pressure	*1.19*	*.47, 1.80*		*1.3*	*.77, 2.18*
Bronchitis/Emphysema	*1.28*	*.65, 2.51*		*2*	*.84, 4.80*
Asthma	*1.11*	*.69, 1.76*		*0.95*	*.51, 1.77*
Allergies	*1.09*	*.56, 2.13*		*0.89*	*.31, 2.53*
Stomach/digestive	*1.06*	*.63, 1.77*		*1.18*	*.59, 2.37*
Liver	*1.06*	*.53, 2.12*		*0.68*	*.29, 1.58*
Bowel/colon	*1.54*	*.90, 2.62*		*1.64*	*.81, 3.32*
Bladder	*1.35*	*.73, 2.51*		*0.76*	*.35, 1.64*
Arthritis	*.59**	*.40, .88*		*.54**	*.30, .95*
Bone, back, joint, muscle	*.76*	*.52, 1.10*		*0.89*	*.53, 1.49*
Gout	*.46*	*.12, 1.83*		*0.57*	*.06, 5.40*
Skin	*.62*	*.35, 1.09*		*0.49*	*.23, 1.04*
Other	*.93*	*.57, 1.51*		*1.06*	*.60, 1.88*
Mental health comorbidity					
No conditions					
Mental health condition(s)	-	-		*1.46*	*.67, 3.16*
Physical health condition(s)	-	-		*0.78*	*.45, 1.36*
Physical & mental health condition(s)	-	-		*1.02*	*.49, 2.10*
Side effects					
No medication					
Never bothers	*1.47**	*1.02, 2.12*		*1.25*	*.77, 2.03*

Bothers a little	*2.93***	*1.35, 6.36*		*1.72*	*.64, 4.67*
Bothers somewhat	*2.31***	*1.23, 4.34*		*0.83*	*.37, 1.86*
Bothers a lot	*2.64***	*1.37, 5.10*		*0.72*	*.25, 1.93*

Alcohol consumption					
Moderate (<14 units)					
None (0 units)	*.63***	*.46, .87*		*.61**	*.42, .90*
Heavy (14-28 units)	*.77*	*.45, 1.34*		*0.53*	*.23, 1.21*
Very heavy (>28 units)	*.51*	*.25, 1.02*		*0.66*	*.31, 1.39*
Smoking status					
Never					
Past occasional smoking	*1.52*	*.88, 2.65*		*1.65*	*.82, 3.32*
Past daily smoking	*1.24*	*.81, 1.90*		*1.05*	*.62, 1.77*
Current occasional smoking	*1.99**	*1.04, 3.81*		*1.78*	*.80, 3.96*
Current daily smoking	*1.92****	*1.35, 2.74*		*1.51*	*.98, 2.33*
Psychological factors					
Empathy	*0.82**	*.70, .96*		*.72***	*.59, .88*
Self-esteem	*.81****	*.75, .88*		*.97*	*.87, 1.09*
Hopelessness	*1.20**	*1.02, 1.41*		*.93*	*.75, 1.15*
Locus of control (power)	*1.08*	*.90, 1.30*		*.89*	*.71, 1.11*
Locus of control (chance)	*1.35***	*1.11, 1.64*		*1.23*	*.95, 1.60*
Locus of control (internal)	*.95*	*.76, 1.19*		*1.05*	*.82, 1.36*
Social capital					
Practical support	*.70*	*.30, 1.61*		*.67*	*.26, 1.70*
Socialise	*1.28*	*.63, 2.61*		*1.57*	*.63, 3.92*
Neighbourhood Belonging	*.69**	*.48, .97*		*.90*	*.58, 1.38*

* $p < .05$, ** $p < .01$, *** $p < .001$
