# Supplementary material for: Investigation to identify individual socioeconomic and health determinants of suicidal ideation using responses to a cross-sectional, community-based public health survey
Source: BMJ Open. 2021 Feb 4;11(2):e035252. doi: 10.1136/bmjopen-2019-035252 (PMC7868260; doi:10.1136/bmjopen-2019-035252)
Supplement: Author's manuscript [file bmjopen-2019-035252.draft_revisions.pdf]

BMJ Open

BMJ Open is committed to open peer review. As part of this commitment we make the peer review history of every article we publish publicly available.

When an article is published we post the peer reviewers' comments and the authors' responses online. We also post the versions of the paper that were used during peer review. These are the versions that the peer review comments apply to.

The versions of the paper that follow are the versions that were submitted during the peer review process. They are not the versions of record or the final published versions. They should not be cited or distributed as the published version of this manuscript.

BMJ Open is an open access journal and the full, final, typeset and author-corrected version of record of the manuscript is available on our site with no access controls, subscription charges or pay-per-view fees (http://bmjopen.bmj.com).

If you have any questions on BMJ Open's open peer review process please email info.bmjopen@bmj.com

BMJ Open

Exploring the socio-economic & health predictors of suicidal ideation in the community

Journal:	*BMJ Open*
Manuscript ID	bmjopen-2019-035252
Article Type:	Original research
Date Submitted by the Author:	28-Oct-2019
Complete List of Authors:	Mulholland, Helen; University of Liverpool, Health Service Research<br>McIntyre, Jason; Liverpool John Moores University, Natural Sciences and Psychology<br>Haines, Alina; Manchester Metropolitan University, Department of Nursing<br>Whittington, Richard; Norwegian University of Science and Technology, Department of Mental Health<br>Comerford, Terence; University of Liverpool, National Institute for Health Research Collaboration for Leadership in Applied Health Research and Care – North West Coast (NIHR CLAHRC-NWC<br>Corcoran, Rhiannon; University of Liverpool, Department of Psychological Sciences
Keywords:	Suicide & self-harm < PSYCHIATRY, MENTAL HEALTH, Adult psychiatry < PSYCHIATRY

SCHOLARONE™
Manuscripts

I, the Submitting Author has the right to grant and does grant on behalf of all authors of the Work (as defined in the below author licence), an exclusive licence and/or a non-exclusive licence for contributions from authors who are: i) UK Crown employees; ii) where BMJ has agreed a CC-BY licence shall apply, and/or iii) in accordance with the terms applicable for US Federal Government officers or employees acting as part of their official duties; on a worldwide, perpetual, irrevocable, royalty-free basis to BMJ Publishing Group Ltd ("BMJ") its licensees and where the relevant Journal is co-owned by BMJ to the co-owners of the Journal, to publish the Work in this journal and any other BMJ products and to exploit all rights, as set out in our licence.

The Submitting Author accepts and understands that any supply made under these terms is made by BMJ to the Submitting Author unless you are acting as an employee on behalf of your employer or a postgraduate student of an affiliated institution which is paying any applicable article publishing charge ("APC") for Open Access articles. Where the Submitting Author wishes to make the Work available on an Open Access basis (and intends to pay the relevant APC), the terms of reuse of such Open Access shall be governed by a Creative Commons licence – details of these licences and which Creative Commons licence will apply to this Work are set out in our licence referred to above.

Other than as permitted in any relevant BMJ Author's Self Archiving Policies, I confirm this Work has not been accepted for publication elsewhere, is not being considered for publication elsewhere and does not duplicate material already published. I confirm all authors consent to publication of this Work and authorise the granting of this licence.

For peer review only

TITLE PAGE

Title:

Exploring the socio-economic & health predictors of suicidal ideation in the community.

Corresponding Author:

Helen Mulholland. 1st Floor, Block B, Waterhouse Building. Department of Health Services Research. The Institute of Population Health Sciences. University of Liverpool. Liverpool. L69 3GL. Email: helen.mulholland@liverpool.ac.uk Telephone: +44 (0)151 795 5323.

Co-Authors:

Dr Jason McIntyre. Natural Sciences and Psychology. Liverpool John Moores University. Liverpool. England.

Dr Alina Haines. Department of Nursing. Manchester Metropolitan University. Manchester. England.

Professor Richard Whittington. Department of Mental Health. Norwegian University of Science and Technology. Trondheim. Norway.

Mr Terence Comerford. National Institute for Health Research Collaboration for Leadership in Applied Health Research and Care – North West Coast (NIHR CLAHRC-NWC). University of Liverpool. Liverpool. England.

Professor Rhiannon Corcoran. Department of Psychological Sciences. University of Liverpool. Liverpool. England.

Word Count: 3,086

For peer review only

For peer review only - http://bmjopen.bmj.com/site/about/guidelines.xhtml

ABSTRACT

Background

Suicidal thoughts and behaviour, are significant public health issues both globally and nationally with severe personal, societal and economic repercussions. The novelty of this study was being able to combine a broad spectrum of survey responses to identify potential predictors of suicidal ideation (SI) across a two week timeframe, within a community based population.

Methods

4319 people from across high (n=20) and low (n=8) deprivation neighbourhoods across the North West of England were recruited via random area probability sampling to participate in a comprehensive public health survey. One resident per household were asked to complete measures comprising demographic, socio-economic, housing and neighbourhood quality, mental health, physical health, wellbeing, lifestyle and social capital factors. Logistic regression analysis was employed to assess predictors of SI.

Results

454 (11%) participants reported having SI within the last 2 weeks. Model 1 (excluding mental health variables) concluded younger age, comorbid physical and mental ill-health, and current smoker status as key predictors of suicidal ideation. Additional statistically significant predictors were noted. Higher self-esteem and neighbourhood belonging as well as alcohol abstinence and unexpectedly, having arthritis, were protective of suicidal ideation. Model 2 (including mental health variables) concluded depression and having cancer as key predictors for suicide ideation. Additional statistically significant predictors were again noted. Alcohol abstinence, having arthritis and higher empathy levels were protective from suicidal ideation the latter being a novel finding.

Conclusion

Interventions should focus upon reducing depression in individuals with suicidal ideation and enhancing their self-esteem, social capital and empathy.

Strengths and limitations of this study

- The novelty of this study was simultaneously investigating a broad spectrum of risk and protective factors and health and social inequalities underpinning suicidal ideation.
- Participants represented a large, non-clinical, community sample.
- Participants were recruited from a wide geographical area incorporating highly deprived and less deprived neighbourhoods, thereby enhancing generalisability of findings.
- This Study utilised a two week measurement timeframe which is relatively novel within suicide research but in line with clinical risk management practices.
- The large sample size mitigates assessing suicidal thoughts using a single item measure.

For peer review only - http://bmjopen.bmj.com/site/about/guidelines.xhtml

For peer review only

MAIN TEXT

BACKGROUND

Suicidal thoughts and behaviour, including suicidal ideation (SI), plans, attempts or self-harm, are significant public health issues both globally and nationally with severe personal, societal and economic repercussions. Suicide is a leading cause of death worldwide whilst SI, attempts and self-harm are strong predictors of suicide deaths and have similar negative health, social and economic consequences[1]. Effective SI and behaviour prevention and clinical risk management strategies are therefore key cross-national priorities.

In support of these priorities much research has been undertaken to better understand SI and behaviour, including underlying risk and protective factors. Risk factors specific to SI have been identified in previous research, e.g. female gender, younger age, lower education and income, unmarried status, unemployment, parent psychopathology, childhood adversities, the presence of an assessed mental disorder, and psychiatric comorbidity[2].

Although theory, research and policies suggest numerous personal and environmental risk and protective factors relating separately to SI and suicide behaviours, single studies that have simultaneously assessed a broad spectrum of individual socio-economic and health determinants of SI using validated measures in large representative samples, remain elusive.

Using responses to a Household Health Survey, this study aimed to conduct exploratory analyses assessing a broad spectrum of variables to identify potential predictors of SI across a two week timeframe. Demographic, socio-economic, housing and neighbourhood quality, mental health, physical health, wellbeing, lifestyle and social capital domains were explored. The dataset thus allowed the examination of understudied phenomena in the suicide literature, such as housing quality and caring responsibilities, alongside specific health conditions. This will shed light on the role of novel determinants of SI, and whether they predict SI over and above the effects of known risk-factors, such as mental health problems, multi-morbidity and

For peer review only - http://bmjopen.bmj.com/site/about/guidelines.xhtml

economic adversity. The results could inform both policy and practice/clinical risk management.

METHODS

Participants and sampling procedure

A cross-sectional public health survey was conducted in the north west of England as part of the National Institute for Health Research Collaboration for Leadership in Applied Health Research and Care – North West Coast (NIHR CLAHRC-NWC). A random area probability sampling strategy was adopted. Twenty high deprivation neighbourhoods and eight less deprived neighbourhoods were selected, and random addresses were contacted within those neighbourhoods. The areas were selected in consultation with local authority representatives based on the following considerations: population size (5,000-10,000 people), level of disadvantage (as measured via Index of Multiple Deprivation), coherent shared identity, and available infrastructure for policy delivery. Overall, 4319 people were recruited between August 2015 and January 2016. There were 809 participants from low deprivation neighbourhoods and 3510 from high deprivation neighbourhoods. This was consistent with the sampling strategy, which had higher targets for high deprivation areas due to the overall project focus of health inequalities. The sample comprised 1854 (43%) men and 2465 (57%) women with ages ranging from 18 to 95 years (M = 49.12, SD = 19.13). The majority of participants (89%) indicated that they were from White European ethnic backgrounds. Participants were reimbursed with a £10 voucher in return for their participation. The adjusted response rate was 61%. See McIntyre et al[3] for a more detailed description of the sampling method and neighbourhood selection procedures.

The research was approved by University of Liverpool Committee on Research Ethics (Ref: RETH00836 and IPHS-1516-SMC-192) and conforms to the principles embodied in the Declaration of Helsinki. Written informed consent was obtained from all participants.

For peer review only - http://bmjopen.bmj.com/site/about/guidelines.xhtml

Measures

For detailed information on each measure and the coding and source of each measure, see the Supplementary File, including Supplementary File Table 1. Descriptive statistics for each measure are captured in Supplementary File Table 2.

Data analysis strategy and preliminary analyses

Data were analysed using Stata version 12[4]. As the dependent variable, suicidal ideation (SI), was highly skewed ($S\text{-}W = .92$, $p < .00001$), the variable was re-coded into 0 = *suicidal ideation absent*, 1 = *suicidal ideation present*. While dichotomization of variables results in potential reductions in effect sizes and power, as well as loss of information, it is recommended for instances of severely skewed data with a large number of participants falling at the extreme end of a scale as we have here[5]. Specifically, 89% (n = 3833) of the sample reported having no SI over the previous 2 weeks, while 454 participants reported having SI.

Given the possibility of collinearity between the four mental health symptoms and between mental health symptoms and SI, Pearson's product moment and Pearson's point-biserial correlations were conducted to examine bivariate relationships. As shown in Table 1, all predictors were moderately correlated with the criterion. The strongest association was between depression and SI, r_{pb} (4285) = .57, $p < .001$. When examining collinearity between predictors, anxiety and depression were highly significantly positively correlated, r (4303) = .79, $p < .001$. As the correlation was below .8 and anxiety and depression represent distinct theoretical constructs, multicollinearity was not considered problematic for the logistic regression analysis[6].

Two logistic regression (LR) analyses were conducted with SI regressed on the socioeconomic, health and lifestyle variables. Standard errors were adjusted to account for the clustered nature of the data using the *svyset* command and the 28 neighbourhoods as clusters.

For peer review only - http://bmjopen.bmj.com/site/about/guidelines.xhtml

The data was also weight-adjusted to account for demographic variation in non-response. The models provided estimates of the odds ratio (OR) of SI associated with each variable, while holding all other variables in the model constant. Because the mental health symptoms explain a substantial portion of variance in SI, we constructed models both including and excluding symptoms to quantify the association between social and health factors and SI, as well as their predictive power above and beyond the effects of mental health. Model 1 excludes, while Model 2 includes mental health symptom variables. Listwise deletion was used to account for missing values in each analysis, which resulted in *n* = 3944 for model 1 and *n* = 3940 for model 2. Missing values analysis indicated that no variable was missing more than 5% of values.

Table 1. *Bivariate correlations between mental health variables*

Variable	1	2	3	4	5
1. Suicidal ideation	-	.57***	.51***	.34***	-.34***
2. Depression	-	-	.79***	.50***	-.52***
3. Anxiety	-	-	-	.55***	-.52***
4. Paranoia	-	-	-	-	-.39***
5. Wellbeing	-	-	-	-	-

*** $p < .001$

For peer review only - http://bmjopen.bmj.com/site/about/guidelines.xhtml

RESULTS

Model 1: Logistic regression predicting suicidal ideation without adjusting for mental health variables

The overall model was significant, $F(65, 3852) = 6.81$, $p < .0001$. Significant effects with alpha set to .001, .01, and .05 are highlighted and adjusted odds ratios are reported alongside confidence intervals within Table 2 below, whilst both significant and non-significant effects for all variables are reported within Supplementary File Table 3. Age was a significant predictor of SI. All younger age groups reported significantly higher odds of SI compared to the base category of 65+ years. Eighteen to twenty-four year olds had the highest increase in odds of SI relative to the base category. People from BME backgrounds had significantly higher odds of SI compared to people from white European backgrounds. Living in lower quality housing was also significantly associated with higher odds of SI. No other socioeconomic variables were associated with SI.

Experiencing moderate or extreme pain/discomfort increased the odds of SI. Of the physical health condition variables, having epilepsy, a stroke or a hearing condition in the previous 12 months was associated with significantly increased odds of SI. Reporting arthritis was associated with significantly lower odds of SI. Having at least one physical and mental health condition significantly increased odds of SI by a factor of 3.31 relative to having no conditions. Having only a mental health condition/s was associated with 3.26 higher odds of SI.

Examination of the psychological risk-factors of mental illness revealed that each 1 unit increase on the single-item self-esteem scale was significantly associated with a 16% lower odds of SI. Conversely, each 1 unit increase on the locus of control chance subscale was significantly associated with 1.34 higher odds of SI.

For peer review only - http://bmjopen.bmj.com/site/about/guidelines.xhtml

Lifestyle factors were also significantly associated with SI. Being a current occasional or heavy smoker was associated with higher odds of SI. Abstaining from alcohol reduced odds of SI by 38% relative to drinking within the recommended limits.

Of the social capital variables, neighbourhood belonging was the only significant predictor. Specifically, every one unit increase in sense of belonging was associated with 33% reduction in odds of SI.

Model 2: Logistic regression predicting suicidal ideation adjusting for mental health variables

The overall model was significant, $F(69, 3844) = 9.38$, $p < .0001$; however, the profile of significant risk factors was somewhat different compared with model 1, as reported with Table 2 below. After adjusting for mental health symptoms, identifying as lesbian, gay, bisexual, queer or transgender (LGBQT) or BME was associated with significantly higher odds of SI. Reporting being in the same financial position as in the previous 12 months was significantly associated with 2.29 times higher odds of SI compared to being in a worse position than 12 months ago,. No other demographic or socioeconomic variables significantly predicted SI.

Reporting a cancer diagnosis was significantly associated with 3.90 higher odds of SI, while reporting arthritis was associated with 46% reduced odds of SI.

Past and present smoking behaviour were unrelated to SI in this model. Abstaining from alcohol was significantly associated with 39% lower odds of SI.

Self-esteem, hopelessness, and locus of control were not associated with SI when adjusting for mental health variables. However, each 1 unit increase in Empathy Quotient scores was associated with a 28% reduction in odds of SI. No social capital variables were associated with SI.

All of the mental health symptoms variables were associated with higher risk of SI. Specifically, anxiety and paranoia were associated with significantly higher odds of SI.

For peer review only - http://bmjopen.bmj.com/site/about/guidelines.xhtml

Depression showed the strongest relationship with SI insofar as each 1 unit increase on the PHQ-9 was associated with 7.25 higher odds of SI. Wellbeing was not related to SI.

Table 2. *Statistically significant logistic regression variables predicting suicidal ideation excluding (Model 1) and including (Model 2) mental health variables.*

	Model 1			*Model 2*	
Predictors	*Adjusted Odds Ratio of suicidal ideation*	*95% CI*		*Adjusted Odds Ratio of suicidal ideation*	*95% CI*
Mental health					
Depression	-	-		*7.24****	*5.22, 10.07*
Anxiety	-	-		*1.56***	*1.13, 2.17*
Paranoia	-	-		*1.36**	*1.72*
Demographics					
Age (65+)					
18-24	*4.43****	*2.20, 8.94*		*0.95*	*.38, 2.38*
25-44	*3.41****	*1.91, 6.10*		*1.62*	*.84, 3.15*
45-64	*2.28***	*1.38, 3.75*		*1.1*	*.60, 2.02*
Black & Minority Ethnic (BME)	*1.94**	*1.11, 3.39*		*1.93**	*1.04, 3.62*
LGBTQ	*2.28*	*.92, 5.66*		*2.73**	*1.00, 7.46*
Socioeconomic status					
Problems with housing	*1.66***	*1.25, 2.21*		*1.34*	*.95, 1.89*
Financial position (worse)					
Same	*1.63*	*.98, 2.69*		*2.29***	*1.24, 4.23*
Health problems (EQ-5D)					
Pain	*1.59**	*1.07, 2.38*		*0.98*	*.61, 1.56*
Health conditions					

For peer review only - http://bmjopen.bmj.com/site/about/guidelines.xhtml

Cancer	*2.16*	*.98, 4.76*	*3.90***	*1.40, 10.84*
Epilepsy	*1.87**	*1.07, 3.28*	*1.65*	*.80, 3.39*
Ear	*2.05**	*1.17, 3.61*	*1.24*	*.59, 2.59*
Stroke	*2.06**	*1.08, 3.95*	*1.63*	*.57, 4.68*
Heart	*0.88*	*.51, 1.51*	*1.3*	*.68, 2.48*
Arthritis	*.58**	*.39, .88*	*.54**	*.30, .95*
Comorbidity				
Mental health condition(s)	*3.26****	*1.85, 5.77*	*1.46*	*.67, 3.16*
Physical & mental health condition(s)	*3.31****	*1.89, 5.79*	*1.02*	*.49, 2.10*
Alcohol consumption				
None (0 units)	*.62***	*.45, .86*	*.61**	*.42, .90*
Smoking status				
Current occasional smoking	*2.01**	*1.03, 3.95*	*1.78*	*.80, 3.96*
Current daily smoking	*1.76***	*1.24, 2.49*	*1.51*	*.98, 2.33*
Psychological factors				
Empathy	*0.86*	*.73, 1.01*	*.72***	*.59, .88*
Self-esteem	*.84****	*.77, .92*	*0.97*	*.87, 1.09*
Locus of control (chance)	*1.34***	*1.10, 1.64*	*1.23*	*.95, 1.60*
Social capital				
Neighbourhood Belonging	*.67**	*.47, .94*	*0.9*	*.58, 1.38*

* $p < .05$, ** $p < .01$, *** $p < .001$

For peer review only

Patient and Public Involvement

Public Advisors were recruited, based upon their personal interest and/or experience of suicide and/or self-harm, from the National Institute of Health Research Collaboration for Leadership in Applied Health Research and Care North West Coast (i.e. NIHR CLAHRC NWC), to participate as Project Team Members for this project. Within this role, the Public Advisors utilised their knowledge and personal experience to influence the research question and survey variables to be included as comparators for the dependent variable of interest. In addition, they

For peer review only - http://bmjopen.bmj.com/site/about/guidelines.xhtml

contributed to the interpretation of the findings following data analysis, as well as project governance. A subgroup of the Public Advisors agreed to review a draft of the manuscript, one of which agreed to be a named co-author.

In addition to the planned peer reviewed publication it is intended that willing Public Advisors will support the dissemination of these findings by providing written and/or oral presentations in line with the NIHR CLAHRC NWC remit. The Public Advisors had an equal voice within the project team, which also comprised academics, researchers and clinicians. The Public Advisors provided positive feedback regarding the formalised structure of the project team meetings (i.e. agenda, minutes, supplementary information) which aided clarity, purpose and participation.

DISCUSSION

Principal findings

This study was uniquely able to combine a broad spectrum of demographic, socio-economic, housing and neighbourhood quality, mental health, physical health, wellbeing, lifestyle and social capital factors to identify potential predictors of suicidal ideation (SI) across a two week timeframe, within a community based population. Given the known significant associations between mental health conditions and suicidal ideation[7], analysis was undertaken both without adjustment and adjusting for mental health variables, in order to examine the individual and combined effects of mental health and other predictive factors.

When not adjusting for mental health factors, younger age (particularly between 18 and 24) and comorbid mental and physical health conditions were the strongest predictors of SI. Being a current occasional smoker doubled people's risk of SI. Other risk factors included: experiencing pain/discomfort; having a stroke or epilepsy; having a hearing condition; being from an ethnic minority background; living in poor quality housing; and having an external locus of control, i.e. believing your life is determined by 'chance' rather than being in your

For peer review only - http://bmjopen.bmj.com/site/about/guidelines.xhtml

control. Protective factors included: alcohol abstinence; higher self-esteem; increased neighbourhood belonging; and surprisingly, suffering from arthritis. However, given that arthritis is more prevalent among older age groups[8], it is plausible that this is partly explained by the strong protective effects of older age.

However, when adjusting for mental health factors, different results emerged. Unsurprisingly, depression was the most important risk factor, whereby those suffering from depression were seven times more likely to report SI than those without. Other risk factors included: having had a cancer diagnosis in the last twelve months; being LGBQT; being in the same (rather than worse) financial position as the previous year; being from an ethnic minority background; suffering from anxiety; and having symptoms of paranoia. Associated protective factors for SI again included alcohol abstinence, suffering from arthritis, and higher empathy.

Depression, anxiety and paranoia were identified here as risk factors for SI, which is congruent with current suicide prevention literature[9].

This study's findings that both mental health and comorbid physical and mental health suggest increased risk of SI also concur with existing evidence. Indeed, physical health multi-morbidities have been shown to correlate with SI both dependently[10] and independently from common mental disorders[11]. Specific long-term conditions that have been previously identified as increasing suicide risk include epilepsy, cancer and coronary heart disease[12]. While no effect of heart conditions on SI was found, having had a cancer diagnosis in the previous 12 months increased the risk of SI by almost four times, independent of mental health and socioeconomic risk factors. This is in line with previous research[13] where cancer was estimated to increase suicide risk by a factor of ten following initial cancer diagnosis. Key determinants of SI identified in the present study including pain/discomfort, cancer, epilepsy and hearing problems may engender perceptions of burdensomeness[14], defeat and entrapment[15],

For peer review only - http://bmjopen.bmj.com/site/about/guidelines.xhtml

psychological and/or physiological pain and/or hopelessness[16], which are all suggested pre-conditions for SI.

Younger age was found to increase SI risk - but only without adjustment for mental health factors, which is again in line with previous literature[2]. Similarly, identifying as LGBQT was linked to SI risk, independently from other factors. However, this finding should be interpreted with caution, given the small number of people falling into this category, large confidence intervals and marginal significance level. Nevertheless, this adds to the evidence from a systematic review of suicide in sexual minority populations which found that suicidal behaviours were more prevalent in young people from sexual minority groups than their heterosexual counterparts[17]. However, sexual orientation did not feature as an independent risk factor when mental health factors were excluded from the analysis, suggesting a potential moderating effect between sexual orientation and mental health factors upon SI.

Thwarted belonging relates to the fundamental need to belong and when compromised can underpin suicidal ideation and behaviour [14]. This may explain the increased risk of SI from minority status groups such as identifying as LGBQT or being from an ethnic minority background, as demonstrated within the results from this study. Further, as the sample was from a predominantly White population, genetic determinants cannot be separated from social confounds such as discrimination and social exclusion experienced by people from BME groups.

Previous research has shown that cancer and dementia patient family caregivers have higher levels of SI with comorbid depression, whilst older age and reasons for living reduce such risks[18]. However, carer status was not found to be a statistically significant predictor here. Carer burden may be exacerbated by longer duration of carer role and particularly challenging patient needs which were not captured within this study and may explain the differences in findings.

For peer review only - http://bmjopen.bmj.com/site/about/guidelines.xhtml

For peer review only

Believing your life to be determined by 'chance' was found to be a risk factor for SI, but only when mental health factors were excluded from the analysis. Given that an external locus of control (LoC) is a known psychological risk factor for psychosis and a proposed mediator of the relationship between social adversity and mental health problems[19], the lack of association in model 2 suggests LoC may mediate the relationship between mental health symptoms and SI. That is, mental health problems may lead people to feel they are not in control of their own lives, which in turn increases risk of SI. Further research with longitudinal data would be required to confirm this hypothesis.

Findings suggesting that alcohol abstinence and higher self-esteem are protective factors for SI are corroborated by previous research[20],[21]. Further, both theory and research confer that higher levels of social capital have a positive impact upon mental health[22] and our analysis excluding mental health factors supports this.

Whilst there is an abundance of research investigating empathy of others towards suicidal individuals, research investigating associations between SI and empathy levels within individuals is limited. This study indicates that higher levels of empathy reduce the risk of SI, without adjustments for mental health factors. Zhang, and colleagues[23] suggest that higher empathy could strengthen social deterrents to SI and behaviour, thereby providing a possible explanation for such a novel association.

Limitations

Whilst based on validated measures with a large representative sample, the survey used in this study is entirely based on self-report. Reporting bias can be an issue, due to the sensitive nature of some of the interview questions. In addition, capturing suicidal ideation (SI) using a single item measure may have resulted in over-simplification, as the validity of single items when detached from a larger instrument is debatable. The large sample size may have mitigated these problems.

For peer review only - http://bmjopen.bmj.com/site/about/guidelines.xhtml

For peer review only

Conclusion

This study was the first to combine a broad spectrum of survey responses to identify potential predictors of SI across a two week timeframe, within a non-clinical, community based population.

These findings support previous research in that minority status, disadvantage, physical/psychological suffering, perceived lack of control, current smoking status, as well as limited coping skills all increase the risk of SI, whilst mental ill-health, in particular depression, has the greatest predictive ability. In contrast, enhanced self-esteem, social capital and empathy were found to be protective factors for SI, the latter being a novel finding within this study. Future research could benefit from exploring this association further. This study investigated respondent perceptions across a two week timeframe. The application of shorter measurement timeframes within suicide research is relatively new, but in line with clinical risk management practices. Generalisability of these findings is enhanced given the mixture of disadvantaged and less disadvantaged areas and random sampling of addresses as well as the application of a statistical adjustment for demographic variation in non-responses. Theoretically, physical health problems, lifestyle factors such as smoking, and living conditions are phenomenon that affect people across many communities and cultures. Thus, these findings have implications for strategies to reduce SI both nationally and internationally.

For peer review only

For peer review only - http://bmjopen.bmj.com/site/about/guidelines.xhtml

COMPETING INTERESTS

Dr McIntyre reports grants from NIHR, during the conduct of the study. Remaining authors have nothing to disclose.

FUNDING

This study is part-funded by The National Institute for Health Research Collaboration for Leadership in Applied Health Research and Care North West Coast (NIHR CLAHRC NWC).

The views expressed here are those of the author(s) and not necessarily those of the NHS, the NIHR, or the Department of Health and Social Care.

AUTHOR CONTRIBUTIONS

The authors have all substantially contributed to this paper.

- HM is the lead author who conceived the study, provided interpretation of the data for this paper and drafted all sections of the manuscript, with the exception of the methods section.
- JMcI has contributed to the design of the study, conducted the data analysis, drafted the methods section and provided substantial critical comments to the whole manuscript at the drafting stage.
- AH has contributed to the design of the study and interpretation of the data, provided substantial critical comments at the drafting stage and conducted final manuscript editing and proofing.
- RW has contributed to the interpretation of the data and provided critical comments at the drafting stage.
- TC has contributed to the design of the study, interpretation of the data and provided critical comments at the drafting stage.
- RC has contributed to the design of the study, interpretation of the data and provided critical comments at the drafting stage.

All authors have approved the final version of the manuscript.

For peer review only - http://bmjopen.bmj.com/site/about/guidelines.xhtml

ACKNOWLEDGMENTS

We would like to acknowledge: Dr Cecil Kullu, Emma Mullin, Elizabeth Fuller, Paula Gross, Jane Shelton, Stuart Wood, and Farheen Yameen for contributing to the design of this project and its’ governance.

EXCLUSIVE LICENCE

I, the Submitting Author has the right to grant and does grant on behalf of all authors of the Work (as defined in the below author licence), an exclusive licence and/or a non-exclusive licence for contributions from authors who are: i) UK Crown employees; ii) where BMJ has agreed a CC-BY licence shall apply, and/or iii) in accordance with the terms applicable for US Federal Government officers or employees acting as part of their official duties; on a worldwide, perpetual, irrevocable, royalty-free basis to BMJ Publishing Group Ltd (“BMJ”) its licensees and where the relevant Journal is co-owned by BMJ to the co-owners of the Journal, to publish the Work in Journal of Epidemiology & Community Health and any other BMJ products and to exploit all rights, as set out in our licence.

The Submitting Author accepts and understands that any supply made under these terms is made by BMJ to the Submitting Author unless you are acting as an employee on behalf of your employer or a postgraduate student of an affiliated institution which is paying any applicable article publishing charge (“APC”) for Open Access articles. Where the Submitting Author wishes to make the Work available on an Open Access basis (and intends to pay the relevant APC), the terms of reuse of such Open Access shall be governed by a Creative Commons licence – details of these licences and which Creative Commons licence will apply to this Work are set out in our licence referred to above

For peer review only

REFERENCES

1. World Health Organisation. Preventing suicide: A global imperative. https://www.who.int/mental_health/suicide-prevention/world_report_2014/en/, 2014.
2. Borges G, Nock MK, Haro Abad JM, et al. Twelve-Month Prevalence of and Risk Factors for Suicide Attempts in the World Health Organization World Mental Health Surveys. *Journal of Clinical Psychiatry* 2010:1617. doi: 10.4088/jcp.08m04967blu
3. McIntyre JC, Wickham S, Barr B, et al. Social Identity and Psychosis: Associations and Psychological Mechanisms. *SCHIZOPHRENIA BULLETIN* 2018;44:681-90. doi: 10.1093/schbul/sbx110
4. Stata Statistical Software: Release 12. College Station, TX: StataCorp LP., 2011.
5. MacCallum RC, Zhang S, Preacher KJ, et al. On the practice of dichotomization of quantitative variables. *Psychological Methods* 2002;7(1):19-40. doi: 10.1037/1082-989X.7.1.19
6. Midi H, Sarkar S, Rana S. Collinearity diagnostics of binary logistic regression model. *Journal of Interdisciplinary Mathematics* 2013;13:253-67. doi: 10.1080/09720502.2010.10700699
7. World Health Organisation. Suicide Key Facts https://www.who.int/en/news-room/fact-sheets/detail/suicide2018 [accessed May 2019.
8. Busija L, Hollingsworth B, Buchbinder R, et al. Role of age, sex, and obesity in the higher prevalence of arthritis among lower socioeconomic groups: A population-based survey. United States: John Wiley & Sons, Ltd, 2007:553.
9. Hawton K, Casañas I Comabella C, Haw C, et al. Risk factors for suicide in individuals with depression: A systematic review. *Journal of Affective Disorders* 2013;147(1-3):17-28. doi: 10.1016/j.jad.2013.01.004
10. Kavalidou K, Smith DJ, O'Connor RC. The role of physical and mental health multimorbidity in suicidal ideation. *Journal of Affective Disorders* 2017;209:80-85. doi: 10.1016/j.jad.2016.11.026
11. Qin P, Webb R, Kapur N, et al. Hospitalization for physical illness and risk of subsequent suicide: A population study. *Journal of Internal Medicine* 2013;273(1):48-58. doi: 10.1111/j.1365-2796.2012.02572.x
12. Department of Health. Preventing suicide in England A cross-government outcomes strategy to save lives https://assets.publishing.service.gov.uk/government/uploads/system/uploads/attachment_data/file/430720/Preventing-Suicide-.pdf, 2012.
13. Webb RT, Kapur N, Kontopantelis E, et al. Suicide risk in primary care patients with major physical diseases: A case-control study. *Archives of General Psychiatry* 2012;69(3):256-64. doi: 10.1001/archgenpsychiatry.2011.1561
14. Joiner TE. Why people die by suicide. Cambridge, MA: Harvard University Press 2005.
15. O'Connor R, Pirkis J. The international handbook of suicide prevention. Second edition. ed: Wiley.
16. Klonsky ED, May AM. Differentiating suicide attempters from suicide ideators: a critical frontier for suicidology research. *Suicide & Life-Threatening Behavior* 2014;44(1):1-5. doi: 10.1111/sltb.12068
17. Yıldız E. Suicide in sexual minority populations: A systematic review of evidence-based studies. *Archives of Psychiatric Nursing* 2018;32(4):650-59. doi: 10.1016/j.apnu.2018.03.003
18. O'Dwyer ST, Moyle W, Zimmer-Gembeck M, et al. Suicidal ideation in family carers of people with dementia. *Aging and Mental Health* 2015:9p. doi: 10.1080/13607863.2015.1063109
19. McIntyre JC, Elahi A, Bentall RP. Social identity and psychosis: Explaining elevated rates of psychosis in migrant populations. *Social and Personality Psychology Compass* 2016;10(11):619-33. doi: 10.1111/spc3.12273
20. Kotrbová K, Dóci I, Hamplová L, et al. Factors Influencing Suicidal Tendencies of Patients with Diagnosis of Attempted Suicide in Medical History and Potential Prevention of Relapse Prevention. *Central European Journal Of Public Health* 2017;25(4):271-76. doi: 10.21101/cejph.a4677

For peer review only - http://bmjopen.bmj.com/site/about/guidelines.xhtml

21. Eades A, Segal DL, Coolidge FL. Suicide Risk Factors Among Older Adults: Exploring Thwarted Belongingness and Perceived Burdensomeness in Relation to Personality and Self-Esteem. *International Journal of Aging and Human Development* 2019;88(2):150-67. doi: 10.1177/0091415018757214
22. Putnam RD. Bowling alone : the collapse and revival of American community: Simon & Schuster 2000.
23. Zhang K, Szanto K, Clark L, et al. Behavioral empathy failures and suicidal behavior. *Behaviour Research and Therapy* 2018 doi: 10.1016/j.brat.2018.10.019

For peer review only

For peer review only - http://bmjopen.bmj.com/site/about/guidelines.xhtml

Supplementary File

Study measures - description

Suicidal ideation (SI)

Suicidal ideation was assessed utilising item 9 of the Patient and Health Questionnaire (PHQ-9[1]). Participants indicated how often over the last two weeks they had been bothered by "thoughts that you would be better off dead, or of hurting yourself in some way". Response options ranged from 1 = *not at all* to 4 = *every day*.

Socio-economic demographics

Participants completed demographic measures, which included age, sex, ethnicity, sexuality, and relationship status. Measures of socioeconomic conditions included education level, employment status, change in financial circumstances over the past year, and housing quality.

Health

Physical health was assessed with the four physical health dimensions of the *EuroQuol five-dimension scale* (EQ-5D[2]). Health conditions were assessed with a single item from the *Adult Psychiatric Morbidity Survey: Survey of Mental Health and Wellbeing, England*[3] that allowed participants to indicate if they suffered from any of 22 named conditions, with the option to list an unnamed condition. Using this item, physical and mental health comorbidity was calculated and categorised into no conditions, mental health condition/s only, physical health condition/s only, and physical and mental health condition/s. Participants also indicated the extent to which medication side effects bothered them. Mental health was assessed using a series of validated instruments. Specifically, depression was measured using the nine-item PHQ-9[1] with item 9 (suicidal ideation) excluded as it was used as the dependent variable; anxiety was measured with the seven-item *Generalised Anxiety Disorder* scale (GAD-7[4]), paranoia was measured using the persecution subscale of the *Persecution and Deservedness Scale* for symptoms of paranoia (PaDS-5[5]), and wellbeing was assessed with the *Warwick-Edinburgh Mental*

For peer review only

Wellbeing Scale (WEMWBS)[6]. Measures of psychological variables known to be associated with poor mental health were also obtained, including the *Single-Item Self Esteem Scale* (SISES[7]), along with abbreviated scales for empathy (*Empathy Quotient*, EQ-Short[8]) hopelessness (*Brief-H-Neg*[9]) and locus of control (Levenson Multidimensional Locus of Control Scales[10]).

Lifestyle factors

Alcohol consumption was measured using the number of units of alcohol participants reported consuming in the previous seven days. Participants were categorised in accordance with recommended drinking guidelines[11] as: abstaining, at or below recommended, above recommended, or more than double recommended levels. Past and present smoking behaviour was assessed with a single item adapted from the NHS Merseyside Lifestyle Survey[12].

Social connectedness/capital

Participants indicated the number of hours they spent caring for a friend or family member. Social support, socialising, and neighbourhood belonging were assessed with single items from the *Community Life Survey* [13].

For peer review only

Supplementary File Table 1. Coding and source of study measures

Variable	Source	Description/Example	Original scoring	Recoding for analysis
Age	Office for National Statistics	N/A	1 = Under 16 years<br>2 = 16-17 years<br>3 = 18-24 years<br>4 = 25-34 years<br>5 = 35-44 years<br>6 = 45-54 years<br>7 = 55-64 years<br>8 = 65-74 years<br>9 = 75+ years	1 = 18-24 years<br>2 = 25-44 years<br>3 = 45-64 years<br>4 = 65+ years
Sex	Office for National Statistics	N/A	1 = Male<br>2 = Female<br>3 = Other	1 = Male<br>2 = Female

For peer review only

Sexuality	Office for National Statistics	N/A	1 = Heterosexual or straight<br>2 = Gay or Lesbian<br>3 = Bisexual<br>4 = Other	0 = heterosexual<br>1 = LGBTQ
Ethnicity	Office for National Statistics	N/A	1 = English / Welsh / Scottish / Northern Irish / British<br>2 = Irish<br>3 = Gypsy or Irish Traveller<br>4 = Any other White background, *please specify*<br>5 = White and Black Caribbean<br>6 = White and Black African<br>7 = White and Asian<br>8 = Any other Mixed / Multiple ethnic background, *please specify*<br>9 = Indian<br>10 = Pakistani<br>11 = Bangladeshi	0 = White<br>1 = BME

For peer review only

			12 = Chinese<br>13 = Any other Asian background, *please specify*<br>14 = African<br>15 = Caribbean<br>16 = Any other Black / African / Caribbean background, *please specify*<br>17 = Arab<br>95 = Any other group, please specify	
Relationship status	Office for National Statistics	N/A	1 = Single/never married or in a same-sex civil partnership<br>2 = Married<br>3 = Separated, but still legally married<br>4 = Divorced<br>5 = Widowed<br>6 = Same-sex civil partnership<br>7 = Separated, but still in a same-sex civil partnership	0 = Partnered<br>1 = Single

For peer review only

			8 = Formerly in a same-sex civil partnership that is now dissolved<br>9 = Surviving partner from a same-sex civil partnership<br>10 = prefer not to say	
Non-employment	Office for National Statistics	N/A	1 = Going to school or college full time (including on vacation)<br>2 = In paid employment or self employed (or temporarily away)<br>3 = On a Government scheme for employment training<br>4 = Doing unpaid work for a business that you own, or that a relative owns<br>5 = Waiting to take up paid work already obtained<br>6 = Looking for paid work or a Government training scheme	0 = employed<br>1 = not employed

For peer review only

			7 = Intending to look for work but prevented by temporary sickness or injury<br>8 = Permanently unable to work because of long-term sickness or disability<br>9 = Retired from paid work<br>10 = Looking after the home or family<br>95 = Doing something else, specify	
Education	Office for National Statistics	Do you have any educational qualifications for which you received a certificate?<br><br>Do you have any professional, vocational or other work-related qualifications for which you received a certificate?	1 = Yes, 2 = No<br><br>1 = Yes, 2 = No	1 = No qualifications<br>2 = Professional or vocational qualification<br>3 = Degree or higher

For peer review only

		What is your highest qualification?	1 = At degree level or above, 2 = Another kind of qualification	
Caring responsibilities	UK Census	Do you look after, or give any help or support to family members, friends, neighbours or others because of either a long term physical or mental ill-health / disability or problems related to old age? Do not count anything you do as part of your paid employment.	1 = No<br>2 = Yes, 1-19 hours/week<br>3 = Yes, 20-49 hours/week<br>4 = Yes, 50+ hours/week	N/A
Housing quality	English Housing Survey	During the winter months, does condensation form on the windows or walls of any room	1 = Yes, 2 = No, 3 = Spontaneous: don't know	0 = No problems<br>1 = One problem<br>2 = Two problems

For peer review only

		in your home apart from the bathrooms or toilets? During the winter months, are there patches of mould or fungus in any room in your home, apart from bathrooms or toilets? During the cold winter weather, can you normally keep comfortably warm in your living room?	1 = Yes, 2 = No, 3 = Spontaneous: don't know<br>1 = Yes, 2 = No, 3 = Spontaneous: don't know	3 = Problems with all three issues
Financial Situation	Wealth and Assets Survey	Would you say your household is better off or worse off	1 = Better off<br>2 = About the same<br>3 = Worse off	1 = Better off<br>2 = About the same<br>3 = Worse off

For peer review only

		financially than you were a year ago?	4 = Don't know	
Physical Health status	EuroQual Five Dimensional Scale (EQ-5D)	Standardised instrument that assesses problems with mobility, self-care, engagement in usual activities, and pain.	1 = No problems<br>2 = Some Problems/Moderate problems<br>3 = Extreme problems/Unable	0 = No problems<br>1 = Some/Severe problems
Physical health conditions	Psychiatric Morbidity Survey	Have you ever had any of [these health conditions] over the past 12 months?	1 = Cancer<br>2 = Diabetes<br>3 = Epilepsy/fits<br>4 = Migraine or other frequent headaches<br>5 = Dementia or Alzheimer's disease<br>6 = Any mental health issue<br>7 = Cataracts / eyesight problems (even if corrected with glasses or contacts)	For each condition:<br><br>0 = condition absent<br>1 = condition present<br><br>For physical and mental health comorbidity:

For peer review only

			8 = Ear/hearing problems (even if corrected with a hearing aid)<br>9 = Stroke<br>10 = Heart attack/angina<br>11 = High blood pressure<br>12 = Bronchitis/emphysema<br>13 = Asthma<br>14 = Allergies<br>15 = Stomach ulcer or other digestive problems<br>16 = Liver problems<br>17 = Bowel/colon problems<br>18 = Bladder problems/incontinences<br>19 = Arthritis<br>20 = Bone, back joint or muscle problems<br>21 = Gout<br>22 = Skin problems	0 = No conditions<br>1 = Mental health condition(s) only<br>2 = Physical health conditions(s) only<br>3 = Physical and mental health condition

For peer review only

			95 = Any other condition	
Side effects	Health Survey for England (HSE)	Do any of your medications cause side effects or bother you in any way?<br><br>If yes, how much does it bothers you?	1 = Yes<br>2 = No<br><br>1 = A lot<br>2 = Somewhat<br>3 = A little<br>4 = Never	0 = No medication<br>1 = Never bothers<br>2 = Bothers a little<br>3 = Bothers somewhat<br>4 = Bothers a lot
Depression	Patient Health Questionnaire (PHQ-9)	Assesses how often participants had been bothered by pro problems such as "Feeling down, depressed, or hopeless" over the past two weeks.	1 = Not at all<br>2 = Several days<br>3 = More than half the days<br>4 = Nearly every day	N/A
Anxiety	Generalised Anxiety	Assesses frequency of events such as "Being so restless that it	1 = Not at all<br>2 = Several days	N/A

For peer review only

	Disorder Questionnaire (GAD-7)	is hard to sit still" over the past two weeks.	3 = More than half the days<br>4 = Nearly every day	
Paranoia	Five-item Persecution and Deservedness Scale (PaDS-5)	Assesses the extent to which people are suspicious of others' intentions. Participants rate their level of agreement with statements such as "I'm often suspicious of other people's intentions towards me."	1 = Strongly disagree<br>2 = Disagree<br>3 = Neither agree or disagree<br>4 = Agree<br>5 = Strongly agree	N/A
Wellbeing	The Warwick-Edinburgh Mental Well-being Scale (WEMWBS)	Abbreviated 7-item scale. Assesses general mood and wellbeing over the previous 2 weeks. Example statements include "I've been feeling	1 = None of the time<br>2 = Rarely<br>3 = Some of the time<br>4 = Often<br>5 = All of the time	N/A

For peer review only

		relaxed" and "I've been thinking clearly".		
Self-esteem	Single-item Self-esteem Scale (SISE)	Validated scale assessing general levels of self-esteem. Participants rate their level of agreement on a 7-point scale with the statement "I have high self-esteem".	1 = Not very true of me<br>7 = Very true of me	
Empathy	Empathy Quotient (EQ)	Abbreviated five-item version. Participants rate their level of agreement with statements such as "I am good at predicting how someone will feel".	1 = Strongly disagree<br>2 = Disagree<br>3 = Neither agree or disagree<br>4 = Agree<br>5 = Strongly agree	
Locus of control	Levenson Locus of Control Scale	Abbreviated nine-item version. Participants rated their level of agreement with statements such	1 = Strongly disagree<br>2 = Disagree<br>3 = Neither agree or disagree	N/A

For peer review only

		as “My life is determined by my own actions”.	4 = Agree<br>5 = Strongly agree	
Alcohol	Merseyside Lifestyle Survey	Participants indicated if they ever drank alcohol and if so how many of the following drinks they had consumed over the past seven days: pints of beer (low, normal and strong), pints of cider, bottles of alcopops, glasses of spirits, glasses of wine (small and large), glasses of fortified wine. These numbers were converted to alcoholic units and then people were categorized into four levels of alcohol consumption	1 = none, moderate (<14 units/week),<br>2 = heavy (14-28 units/week),<br>3 = very heavy (>28 units/week).	N/A

For peer review only

		based on the recommended usage of less than 14 units per week (Department of Health, 2016)		
Smoking status	Merseyside Lifestyle Survey	N/A	1 = Never smoked<br>2 = Past occasional smoker<br>3 = Past daily smoker<br>4 = Current occasional smoker<br>5 = Current daily smoker	N/A
Social capital	Community Life Survey	Assesses the extent to which participants agree they receive *practical support* ("If I needed help, there are people who would be there for me") and *social contact* ("If I wanted	1 = Definitely agree<br>2 = Tend to agree<br>3 = Tend to disagree<br>4 = Definitely disagree<br>5 = Don't know	0 = Disagree<br>1 = Agree

For peer review only

		company or to socialise, there are people I can call on”)		
Neighbourhood belonging	Community Life Survey	Participants were asked “how strongly you feel you belong to your immediate neighbourhood”.	1 = Very strongly<br>2 = Fairly strongly<br>3 = Not very strongly<br>4 = Not at all strongly<br>5 = Don’t know	0 = Not very/at all strongly<br>1 = Fairly/very strongly

For peer review only

Supplementary File Table 2. *Descriptive statistics for all variables.*

	N (%)	*M* (*SD*)
**Suicidal ideation in previous 2 weeks**		
Suicidal ideation	454 (9.4%)	-
No suicidal ideation	3833 (90.6)	-
**Mental health symptoms**		
Depression (PHQ-9)	-	4.65(5.77)
Anxiety (GAD-7)	-	9.00 (4.33)
Paranoia (PaDS-5)	-	9.68 (4.38)
**Age**		
18-24 years	421 (9.8)	-
25-44 years	1438 (33.3)	-
45-64 years	1329 (30.8)	-
65+ years	1129 (26.2)	-
**Gender**		
Female	2465 (57.1)	-
Male	1854 (42.9)	-
**Ethnicity**		
Black and Minority Ethnic	455 (10.6)	-
White	3855 (89.4)	-
**Sexuality**		
LGBTQ	65 (1.5)	-
Not LGBTQ	4246 (98.5)	-
**Relationship status**		
Not married/civil partnership	2559 (49.4)	-
Married/civil partnership	1747 (40.6)	-
**Education**		
No qualifications	1718 (39.9)	-
Professional/vocational certificate	1931 (44.8)	-
Degree or higher	659 (15.3)	-
**Employment status**		
Employed	1745 (40.4)	-
Not employed	2570 (59.6)	-

For peer review only

**Housing quality (0-1 scale)**	-	.32 (.47)
**Financial position compared with 12 months ago**		
Worse	679 (15.7)	-
Same	3090 (72.2)	-
Better	510 (11.8)	-
**Caring responsibilities**		
None	3716 (86.0)	-
1-19 hours/week	275 (6.4)	-
20-49 hours/week	100 (2.3)	-
50+ hours/week	228 (5.3)	-
**Health problems (EQ-5D; 0 – 1 scale)**		
Mobility	-	.24 (.43)
Self-care	-	.10 (.29)
Usual activities	-	.22 (.42)
Pain	-	.36 (.48)
**Health conditions**		
Cancer	120 (2.8)	-
Diabetes	341 (7.9)	-
Epilepsy	80 (1.9)	-
Migraine	348 (8.1)	-
Dementia	21 (0.5)	-
Eye	418 (9.7)	-
Ear	247 (5.7)	-
Stroke	97 (2.3)	-
Heart	280 (6.5)	-
Blood pressure	711 (16.5)	-
Bronchitis/Emphysema	113 (2.6)	-
Asthma	418 (9.7)	-
Allergies	185 (4.3)	-
Stomach/digestive	224 (5.2)	-
Liver	73 (1.7)	-
Bowel/colon	173 (4.0)	-
Bladder	136 (3.2)	-
Arthritis	728 (16.9)	-
Bone, back, joint, muscle	778 (18.0)	-
Gout	51 (1.2)	-
Skin	243 (5.6)	-
Other	267 (6.2)	-
**Side effects**		
No medication	2015 (46.8)	-
Never bothers	1945 (45.2)	-

For peer review only

Bothers a little	79 (1.8)	-
Bothers somewhat	145 (3.4)	-
Bothers a lot	125 (2.9)	-
**Alcohol consumption**		
Never (0 units/week)	1894 (44.0)	-
Moderate (1-14 units/week)	1973 (45.8)	-
High (14-28 units/week)	290 (6.7)	-
Very high (>28 units/week)	151 (3.5)	-
**Smoking status**		
Never	2107 (48.8)	-
Past occasional smoking	286 (6.6)	-
Past daily smoking	671 (15.5)	-
Current occasional smoking	122 (2.8)	-
Current daily smoking	1118 (25.9)	-
**Psychological factors**		
Empathy	-	3.35 (.88)
Self-esteem	-	4.54 (1.73)
Hopelessness	-	2.48 (.97)
Locus of control (power)	-	2.83 (.88)
Locus of control (chance)	-	2.76 (.74)
Locus of control (internal)	-	3.65 (.69)
**Social capital and belonging**		
Practical support	4084 (94.8)	-
People to socialise with	4064 (94.4)	-
Neighbourhood belonging	3518 (82.1)	-

For peer review only

Supplementary File Table 3. *Logistic regression predicting suicidal ideation excluding (Model 1) and including (Model 2) mental health variables.*

	Model 1			*Model 2*	
Predictors	*Adjusted Odds Ratio of suicidal ideation*	*95% CI*		*Adjusted Odds Ratio of suicidal ideation*	*95% CI*
Mental health					
Depression	-	-		*7.24****	*5.22, 10.07*
Anxiety	-	-		*1.56***	*1.13, 2.17*
Paranoia	-	-		*1.36**	*1.72*
Wellbeing	-	-		*0.76*	*.56, 1.01*
Demographics					
Age (65+)					
18-24	*4.43****	*2.20, 8.94*		*0.95*	*.38, 2.38*
25-44	*3.41****	*1.91, 6.10*		*1.62*	*.84, 3.15*
45-64	*2.28***	*1.38, 3.75*		*1.1*	*.60, 2.02*
Female	*0.91*	*.68, 1.21*		*0.81*	*.57, 1.14*
Black & Minority Ethnic (BME)	*1.94**	*1.11, 3.39*		*1.93**	*1.04, 3.62*
LGBTQ	*2.28*	*.92, 5.66*		*2.73**	*1.00, 7.46*
Single/never married or civil partnership	*1.12*	*.82, 1.52*		*1.07*	*.74, 1.56*

For peer review only

Socioeconomic status					
Education (No qualifications)					
Professional, vocational or work Certificate	*0.96*	*.70, 1.33*		*0.76*	*.52, 1.12*
Degree or higher	*0.94*	*.60, 1.47*		*0.7*	*.40, 1.21*
Non-employment	*1.22*	*.86, 1.74*		*1.06*	*.68, 1.65*
Problems with housing	*1.66*****	*1.25, 2.21*		*1.34*	*.95, 1.89*
Financial position (worse)					
Same	*1.63*	*.98, 2.69*		*2.29*****	*1.24, 4.23*
Better	*1.58*	*.89, 2.78*		*1.19*	*.58, 2.42*
Caring responsibilities					
None					
1-19 hours/week	*0.69*	*.38, 1.28*		*0.64*	*.32, 1.30*
20-49 hours/week	*1.36*	*.69, 2.71*		*1*	*.39, 2.57*
50+ hours/week	*1.12*	*.60, 2.07*		*0.68*	*.31, 1.49*
Health problems (EQ-5D)					
Pain	*1.59**	*1.07, 2.38*		*0.98*	*.61, 1.56*
Self-care	*1.4*	*.84, 2.34*		*1.02*	*.50, 2.08*
Usual activities	*0.94*	*.59, 1.50*		*0.64*	*.35, 1.18*
Mobility	*1.07*	*.68, 1.68*		*1.12*	*.65, 1.93*
Health conditions					
Cancer	*2.16*	*.98, 4.76*		*3.90*****	*1.40, 10.84*
Diabetes	*1.13*	*.62, 2.05*		*0.86*	*.43, 1.75*
Epilepsy	*1.87**	*1.07, 3.28*		*1.65*	*.80, 3.39*
Migraine	*1.08*	*.68, 1.70*		*0.73*	*.39, 1.35*
Dementia	*0.94*	*.33, 2.69*		*0.29*	*.06, 1.47*
Eye	*0.94*	*.60, 1.47*		*1.23*	*.74, 2.06*
Ear	*2.05**	*1.17, 3.61*		*1.24*	*.59, 2.59*

For peer review only

Stroke	*2.06**	*1.08, 3.95*		*1.63*	*.57, 4.68*
Heart	*0.88*	*.51, 1.51*		*1.3*	*.68, 2.48*
Blood pressure	*1.23*	*.81, 1.88*		*1.3*	*.77, 2.18*
Bronchitis/Emphysema	*1.18*	*.58, 2.41*		*2*	*.84, 4.80*
Asthma	*1.15*	*.71, 1.85*		*0.95*	*.51, 1.77*
Allergies	*1.02*	*.49, 2.13*		*0.89*	*.31, 2.53*
Stomach/digestive	*1.03*	*.61, 1.74*		*1.18*	*.59, 2.37*
Liver	*0.96*	*.45, 2.05*		*0.68*	*.29, 1.58*
Bowel/colon	*1.47*	*.85, 2.56*		*1.64*	*.81, 3.32*
Bladder	*1.22*	*.63, 2.35*		*0.76*	*.35, 1.64*
Arthritis	*.58**	*.39, .88*		*.54**	*.30, .95*
Bone, back, joint, muscle	*0.75*	*.52, 1.10*		*0.89*	*.53, 1.49*
Gout	*0.58*	*.14, 2.40*		*0.57*	*.06, 5.40*
Skin	*0.6*	*.33, 1.09*		*0.49*	*.23, 1.04*
Other	*0.97*	*.58, 1.62*		*1.06*	*.60, 1.88*
Comorbidity					
No conditions					
Mental health condition(s)	*3.26****	*1.85, 5.77*		*1.46*	*.67, 3.16*
Physical health condition(s)	*1.01*	*.64, 1.60*		*0.78*	*.45, 1.36*
Physical & mental health condition(s)	*3.31****	*1.89, 5.79*		*1.02*	*.49, 2.10*
Side effects					
No medication					
Never bothers	*1.13*	*.76, 1.68*		*1.25*	*.77, 2.03*
Bothers a little	*2.07*	*.90, 4.73*		*1.72*	*.64, 4.67*
Bothers somewhat	*1.51*	*.81, 2.80*		*0.83*	*.37, 1.86*
Bothers a lot	*1.67*	*.83, 3.36*		*0.72*	*.25, 1.93*

For peer review only

Alcohol consumption					
Moderate (<14 units)					
None (0 units)	*.62***	*.45, .86*		*.61**	*.42, .90*
Heavy (14-28 units)	*0.8*	*.46, 1.39*		*0.53*	*.23, 1.21*
Very heavy (>28 units)	*0.5*	*.24, 1.05*		*0.66*	*.31, 1.39*
Smoking status					
Never					
Past occasional smoking	*1.55*	*.88, 2.73*		*1.65*	*.82, 3.32*
Past daily smoking	*1.27*	*.82, 1.98*		*1.05*	*.62, 1.77*
Current occasional smoking	*2.01**	*1.03, 3.95*		*1.78*	*.80, 3.96*
Current daily smoking	*1.76***	*1.24, 2.49*		*1.51*	*.98, 2.33*
Psychological factors					
Empathy	*0.86*	*.73, 1.01*		*.72***	*.59, .88*
Self-esteem	*.84****	*.77, .92*		*0.97*	*.87, 1.09*
Hopelessness	*1.17*	*.99, 1.38*		*0.93*	*.75, 1.15*
Locus of control (power)	*1.1*	*.91, 1.32*		*0.89*	*.71, 1.11*
Locus of control (chance)	*1.34***	*1.10, 1.64*		*1.23*	*.95, 1.60*
Locus of control (internal)	*0.95*	*.75, 1.19*		*1.05*	*.82, 1.36*
Social capital					
Practical support	*0.66*	*.28, 1.54*		*0.67*	*.26, 1.70*
Socialise	*1.5*	*.73, 3.09*		*1.57*	*.63, 3.92*
Neighbourhood Belonging	*.67**	*.47, .94*		*0.9*	*.58, 1.38*

* $p < .05$, ** $p < .01$, *** $p < .001$

References

1. Kroenke K, Spitzer RL. The PHQ-9: A New Depression Diagnostic and Severity Measure The nine-item Patient Health Questionnaire depression scale is a dual-purpose instrument that can establish provisional depressive disorder diagnoses as well as grade depression severity. United States: SLACK INCORPORATED, 2002:509.
2. Gusi N, Olivares P, Rajendram R. The EQ-5D health-related quality of life questionnaire. *Handbook of disease burdens and quality of life measures* 2010:87-99.
3. McManus S, Bebbington P, Jenkins R, et al. Adult psychiatric morbidity survey: survey of mental health and wellbeing, England, 2014: NHS Digital Leeds, UK, 2016.
4. Spitzer RL, Kroenke K, Williams JBW, et al. A brief measure for assessing generalized anxiety disorder: The GAD-7. *Archives of Internal Medicine* 2006;166(10):1092-97. doi: 10.1001/archinte.166.10.1092
5. Melo S, Corcoran R, Shryane N, et al. The persecution and deservedness scale. *Psychology & Psychotherapy: Theory, Research & Practice* 2009;82(3):247-60. doi: 10.1348/147608308X398337
6. Tennant R, Hiller L, Fishwick R, et al. The Warwick-Edinburgh Mental Well-being Scale (WEMWBS): development and UK validation. *Health And Quality Of Life Outcomes* 2007;5:63-63.
7. Robins RW, Hendin HM, Trzesniewski KH. Measuring Global Self-Esteem: Construct Validation of a Single-Item Measure and the Rosenberg Self-Esteem Scale. *Personality & Social Psychology Bulletin* 2001(2)
8. Baron-Cohen S, Wheelwright S. The Empathy Quotient: An Investigation of Adults with Asperger Syndrome or High Functioning Autism, and Normal Sex Differences. *Journal of Autism and Developmental Disorders* 2004(2):163.
9. Everson SA, Goldberg DE, Kaplan GA, et al. Hopelessness and Risk of Mortality and Incidence of Myocardial Infarction and Cancer. *Psychosomatic Medicine* 1996;58(2):113-21. doi: 10.1097/00006842-199603000-00003
10. Levenson H. Multidimensional locus of control in psychiatric patients. *Journal of Consulting and Clinical Psychology* 1973;41(3):397-404. doi: 10.1037/h0035357
11. Department of Health. UK Chief Medical Officers' low risk drinking guidelines: Department of Health London, 2016.
12. Knowsley Council. NHS Merseyside Lifestyle Survey. http://www.knowsley.gov.uk/pdf/knowsley-health-and-lifestyle-survey-2012-13.pdf, 2013.
13. United Kingdom Cabinet Office. Community Life Survey 2014-15. https://www.gov.uk/government/publications/community-life-survey-2014-to-2015-statistical-analysis, 2015.

For peer review only

Reporting checklist for cross sectional study.

Based on the STROBE cross sectional guidelines.

Instructions to authors

Complete this checklist by entering the page numbers from your manuscript where readers will find each of the items listed below.

Your article may not currently address all the items on the checklist. Please modify your text to include the missing information. If you are certain that an item does not apply, please write "n/a" and provide a short explanation.

Upload your completed checklist as an extra file when you submit to a journal.

In your methods section, say that you used the STROBE cross sectionalreporting guidelines, and cite them as:

von Elm E, Altman DG, Egger M, Pocock SJ, Gotzsche PC, Vandenbroucke JP. The Strengthening the Reporting of Observational Studies in Epidemiology (STROBE) Statement: guidelines for reporting observational studies.

		Reporting Item	Page Number
**Title and abstract**			
Title	#1a	Indicate the study's design with a commonly used term in the title or the abstract	2
Abstract	#1b	Provide in the abstract an informative and balanced summary of what was done and what was found	2

For peer review only

Introduction

Background / rationale	#2	Explain the scientific background and rationale for the investigation being reported	3
Objectives	#3	State specific objectives, including any prespecified hypotheses	3

Methods

Study design	#4	Present key elements of study design early in the paper	4
Setting	#5	Describe the setting, locations, and relevant dates, including periods of recruitment, exposure, follow-up, and data collection	4
Eligibility criteria	#6a	Give the eligibility criteria, and the sources and methods of selection of participants.	4
	#7	Clearly define all outcomes, exposures, predictors, potential confounders, and effect modifiers. Give diagnostic criteria, if applicable	4
Data sources / measurement	#8	For each variable of interest give sources of data and details of methods of assessment (measurement). Describe comparability of assessment methods if there is more than one group. Give information separately for for exposed and unexposed groups if applicable.	Supplementary File

For peer review only

Bias	#9	Describe any efforts to address potential sources of bias	5
Study size	#10	Explain how the study size was arrived at	4
Quantitative variables	#11	Explain how quantitative variables were handled in the analyses. If applicable, describe which groupings were chosen, and why	Supplementary File
Statistical methods	#12a	Describe all statistical methods, including those used to control for confounding	5
Statistical methods	#12b	Describe any methods used to examine subgroups and interactions	5
Statistical methods	#12c	Explain how missing data were addressed	6
Statistical methods	#12d	If applicable, describe analytical methods taking account of sampling strategy	5
Statistical methods	#12e	Describe any sensitivity analyses	5
**Results**			
Participants	#13a	Report numbers of individuals at each stage of study—eg numbers potentially eligible, examined for eligibility, confirmed eligible, included in the study, completing follow-up, and analysed. Give information separately for for exposed and unexposed groups if applicable.	4

For peer review only

Participants	#13b	Give reasons for non-participation at each stage	4
Participants	#13c	Consider use of a flow diagram	
Descriptive data	#14a	Give characteristics of study participants (eg demographic, clinical, social) and information on exposures and potential confounders. Give information separately for exposed and unexposed groups if applicable.	Supplementary File
Descriptive data	#14b	Indicate number of participants with missing data for each variable of interest	6
Outcome data	#15	Report numbers of outcome events or summary measures. Give information separately for exposed and unexposed groups if applicable.	
Main results	#16a	Give unadjusted estimates and, if applicable, confounder-adjusted estimates and their precision (eg, 95% confidence interval). Make clear which confounders were adjusted for and why they were included	Supplementary File
Main results	#16b	Report category boundaries when continuous variables were categorized	na
Main results	#16c	If relevant, consider translating estimates of relative risk into absolute risk for a meaningful time period	na
Other analyses	#17	Report other analyses done—e.g., analyses of subgroups and interactions, and sensitivity analyses	6

For peer review only

**Discussion**			
Key results	#18	Summarise key results with reference to study objectives	11
Limitations	#19	Discuss limitations of the study, taking into account sources of potential bias or imprecision. Discuss both direction and magnitude of any potential bias.	14
Interpretation	#20	Give a cautious overall interpretation considering objectives, limitations, multiplicity of analyses, results from similar studies, and other relevant evidence.	15
Generalisability	#21	Discuss the generalisability (external validity) of the study results	15
**Other Information**			
Funding	#22	Give the source of funding and the role of the funders for the present study and, if applicable, for the original study on which the present article is based	16

None The STROBE checklist is distributed under the terms of the Creative Commons Attribution License CC-BY. This checklist can be completed online using https://www.goodreports.org/, a tool made by the EQUATOR Network in collaboration with Penelope.ai

For peer review only

BMJ Open

An investigation to identify individual socio-economic and health determinants of suicidal ideation using responses to a cross-sectional, community-based public health survey.

Journal:	*BMJ Open*
Manuscript ID	bmjopen-2019-035252.R1
Article Type:	Original research
Date Submitted by the Author:	22-Oct-2020
Complete List of Authors:	Mulholland, Helen; University of Liverpool, Primary Care and Mental Health<br>McIntyre, Jason; Liverpool John Moores University, Natural Sciences and Psychology<br>Haines, Alina; Manchester Metropolitan University, Department of Nursing<br>Whittington, Richard; Norwegian University of Science and Technology, Brøset Centre for Research and Education in Forensic Psychiatry, St. Olav's Hospital and Department of Mental Health.<br>Comerford, Terence; University of Liverpool, National Institute for Health Research Collaboration for Leadership in Applied Health Research and Care – North West Coast (NIHR CLAHRC-NWC<br>Corcoran, Rhiannon; University of Liverpool, Primary Care and Mental Health
**Primary Subject Heading**:	Mental health
Secondary Subject Heading:	Health services research, Public health
Keywords:	Suicide & self-harm < PSYCHIATRY, MENTAL HEALTH, Adult psychiatry < PSYCHIATRY

SCHOLARONE™
Manuscripts

I, the Submitting Author has the right to grant and does grant on behalf of all authors of the Work (as defined in the below author licence), an exclusive licence and/or a non-exclusive licence for contributions from authors who are: i) UK Crown employees; ii) where BMJ has agreed a CC-BY licence shall apply, and/or iii) in accordance with the terms applicable for US Federal Government officers or employees acting as part of their official duties; on a worldwide, perpetual, irrevocable, royalty-free basis to BMJ Publishing Group Ltd ("BMJ") its licensees and where the relevant Journal is co-owned by BMJ to the co-owners of the Journal, to publish the Work in this journal and any other BMJ products and to exploit all rights, as set out in our licence.

The Submitting Author accepts and understands that any supply made under these terms is made by BMJ to the Submitting Author unless you are acting as an employee on behalf of your employer or a postgraduate student of an affiliated institution which is paying any applicable article publishing charge ("APC") for Open Access articles. Where the Submitting Author wishes to make the Work available on an Open Access basis (and intends to pay the relevant APC), the terms of reuse of such Open Access shall be governed by a Creative Commons licence – details of these licences and which Creative Commons licence will apply to this Work are set out in our licence referred to above.

Other than as permitted in any relevant BMJ Author's Self Archiving Policies, I confirm this Work has not been accepted for publication elsewhere, is not being considered for publication elsewhere and does not duplicate material already published. I confirm all authors consent to publication of this Work and authorise the granting of this licence.

For peer review only

TITLE PAGE

Title: An investigation to identify individual socio-economic and health determinants of suicidal ideation using responses to a cross-sectional, community-based public health survey.

Corresponding Author:

Helen Mulholland. 1st Floor, Block B, Waterhouse Building. Department of Primary Care and Mental Health. Institute of Population Health. University of Liverpool. Liverpool. England. L69 3GL. Email: helen.mulholland@liverpool.ac.uk Telephone: +44 (0)151 795 5323.

Co-Authors:

Dr Jason McIntyre. Natural Sciences and Psychology. Liverpool John Moores University. Liverpool. England.

Dr Alina Haines-Delmont. Department of Nursing. Manchester Metropolitan University. Manchester. England.

Professor Richard Whittington. Brøset Centre for Research and Education in Forensic Psychiatry, St. Olav's Hospital and Department of Mental Health. Norwegian University of Science and Technology. Trondheim. Norway.

Mr Terence Comerford. The National Institute for Health Research Applied Research Collaboration North West Coast (NIHR ARC NWC). University of Liverpool. Liverpool. England.

Professor Rhiannon Corcoran. Department of Primary Care and Mental Health. Institute of Population Health. University of Liverpool. Liverpool. England.

Word Count: 4706

For peer review only

ABSTRACT

Objectives: To address a gap in knowledge by simultaneously assessing a broad spectrum of individual socio-economic and potential health determinants of suicidal ideation (SI) using validated measures in a large UK representative community sample

Design: In this cross-sectional design, participants were recruited via random area probability sampling to participate in a comprehensive public health survey. The questionnaire examined demographic, health and socio-economic factors. Logistic regression analysis was employed to identify predictors of SI.

Setting: Community setting from high (n=20) and low (n=8) deprivation neighbourhoods across the North West of England, UK.

Participants: 4319 people were recruited between August 2015 and January 2016. There were 809 participants from low deprivation neighbourhoods and 3510 from high deprivation neighbourhoods. The sample comprised 1854 (43%) men and 2465 (57%) women.

Primary outcome measures: SI was the dependent variable which was assessed using item 9 of the PHQ-9 instrument.

Results: 454 (11%) participants reported having SI within the last 2 weeks. Model 1 (excluding mental health variables) identified younger age, black and minority ethnic (BME) background, lower housing quality and current smoker status as key predictors of SI. Higher self-esteem, empathy and neighbourhood belonging, alcohol abstinence and having arthritis, were protective against SI. Model 2 (including mental health variables) found depression and having

For peer review only - http://bmjopen.bmj.com/site/about/guidelines.xhtml

cancer as key health predictors for SI, while identifying as lesbian, gay, bisexual, transgender, or queer (LGBTQ) and BME were significant demographic predictors. Alcohol abstinence, having arthritis and higher empathy levels were protective against SI.

Conclusions: This study suggests that it could be useful to increase community support and sense of belonging using a public health approach for vulnerable groups (e.g. those with cancer) and peer support for people who identify as LGBTQ and/or BME. Also, interventions aimed at increasing empathic functioning may prove effective for reducing SI.

Strengths and limitations of this study

- This study identified a number of novel protective factors associated with suicidal ideation including: neighbourhood belonging, level of self-reported empathy and abstaining from alcohol.
- Participants represented a large, non-clinical, community sample.
- Generalisability of these findings is enhanced given the mixture of disadvantaged and less disadvantaged areas and random sampling of addresses as well as the application of a statistical adjustment for demographic variation in non-responses.
- This study utilised a two-week measurement timeframe which is relatively novel within suicide research but in line with clinical risk management practices.
- The large overall sample size mitigates the limitation of assessing suicidal thoughts using a single item measure.

For peer review only - http://bmjopen.bmj.com/site/about/guidelines.xhtml

MAIN TEXT

BACKGROUND

The eradication of suicide is a key national and global health policy.[1, 2] Approximately nine percent of people, across cultures, will experience suicidal thoughts (i.e. suicidal ideation) at some point in their lifetime.[3] Over a third of these people will plan their suicide, whilst over half of these people who plan will attempt suicide.[3] The personal impact of suicidal ideation has been likened to suffering severe asthma or alcohol dependence.[4] Despite this significant disease burden, suicidal ideation remains largely untreated with just 34% - 42% of people receiving clinical or non-healthcare support.[5] The key reasons for this do not seem to relate to structural factors such as treatment availability. Rather they relate to a low perceived need for treatment by individuals and a preference for personal rather than formal management.[5] These preferences may reflect the historical stigma associated with mental health broadly, and suicide specifically.[1 2]

However, the findings referred to above come from the World Health Organisation (WHO) World Mental Health Surveys[6] which exclude the United Kingdom (UK). Lifetime prevalence of suicidal ideation within the UK population has been estimated to be more than double that of the cross-national prevalence rates.[7] Given the significant prevalence of suicidal ideation and the apparent reticence of individuals to seek formal support, suicidal ideation identification and clinical intervention strategies are imperative both nationally and globally.[1,2]

In support of these priorities, research has focussed upon understanding suicidal ideation, including underlying risk and protective factors. Risk factors specific to suicidal ideation identified in previous research include: female gender, parent psychopathology, childhood adversities, the presence of an assessed mental disorder, and psychiatric comorbidity.[8] However, existing research has been criticised for a narrow focus on factors associated with individuals, while excluding societal and cultural factors, such as relative

For peer review only - http://bmjopen.bmj.com/site/about/guidelines.xhtml

inequalities and relational matters.[9] Indeed, in their systematic review of reviews post 2007, McClatchley and colleagues[9] summarised risk factors for suicide ideation, suicidal behaviours (i.e. suicide attempts) and suicide completion, to include: mental ill-health; physical health (e.g. Traumatic brain injury; Type 1 diabetes mellitus); health behaviours (e.g. smoking; substance use (including alcohol); biopsychosocial factors (e.g. parental suicide); experience of abuse; internet use; cyber bullying; lesbian, gay, and bisexual (LGB) sexuality; unemployment; 'elementary' occupations such as cleaners, agricultural workers; veterinary surgeons; military veterans; and environmental factors; (e.g. access to means). Of note, McClatchley et al.'s review did not elaborate separate risk factors between suicidal ideation and suicidal behaviour.

Current 'ideation-to-action' theories of suicide also describe the complex interplay of biological, psychological, environmental, and cultural factors that influence the inception of suicidal ideation and the progression from suicidal ideation to behaviour, such as the Integrated-Motivational-Volitional (IMV) Model of Suicide[10] and the Three Step Theory.[11] The IMV Model of Suicide[10] suggests that an interplay between background factors such as: personal disposition, deprivation, adversity and negative life events, can generate feelings of defeat and/or humiliation. These feelings endure and underpin perceptions of entrapment when negative appraisals of the personal agency and/or motivation to overcome such defeat/humiliation are experienced, leading to the development of suicidal ideation. This inability to generate and implement positive solutions to personal problems may be due to the processes of cognitive restriction and deconstruction described by Baumeister[12] and / or emotional dysregulation[13] whereby individuals oscillate between emotional sensitivity/reactivity and emotional inhibition.[14] Alternatively, the social determinants of individuals mental and physical health, rather than individual personal agency, may underpin perceptions of entrapment and lack of control. [15]

For peer review only - http://bmjopen.bmj.com/site/about/guidelines.xhtml

Although theory, evidence and policies suggest numerous personal and environmental risk and protective factors relating to suicidal ideation, single studies that simultaneously assess a broad spectrum of individual socio-economic and health determinants of suicidal ideation using validated measures in large representative community samples, are extremely scarce. In one such example, using the Composite International Diagnostic Interview, Handley et al[16] concluded that, younger age, being unmarried, lifetime anxiety or lifetime post-traumatic stress independently predicted suicidal ideation in an Australian rural community sample, after having controlled for lifetime depression.

Using responses to a community-based Household Health Survey (HHS), this study aimed to address this gap in the literature. Exploratory analyses sought to identify wider determinants of suicidal ideation, with a specific focus upon the impacts of health inequalities, to identify potential risk and protective factors specifically pertinent to suicidal ideation experienced across a two-week timeframe. Therefore, a subset of survey responses to demographic, socio-economic, housing and neighbourhood, mental health, physical health, wellbeing, lifestyle and social capital domains were explored. The dataset thus allowed the examination of understudied phenomena in the suicide ideation literature, such as housing quality, caring responsibilities and medication side-effects which may underpin perceptions of entrapment. Indeed, poor housing quality – defined as accommodation with condensation, mould or fungus – has been shown to have a detrimental impact upon both mental and physical wellbeing.[17] Carer burden has been identified as a significant risk, suggesting up to a four-fold increased risk of suicidal ideation among carers across different patient populations, such as HIV,[18] chronic disease,[19] dementia[20] and cancer[21] compared with the general population. The literature regarding the link between medication side effects and suicidal ideation is limited to clinical populations and antipsychotics/antidepressants. Current evidence suggests that 'Treatment-Emergent Suicidal Ideation' (TESI) is relatively uncommon in older depressed

For peer review only - http://bmjopen.bmj.com/site/about/guidelines.xhtml

adults.[22]. One study exploring the psychiatric side effects of Chloroquine and Hydroxychloroquine found some evidence suggesting a weak positive association with suicidal ideation.[23] In addition, measures of empathy and social capital may act as protective motivational moderators between perceptions of entrapment and the development of suicide ideation, representing greater conformity to social norms/attitudes and perceived social belonging and/or support, also described in the IMV model of suicide.[10] Indeed, Zhang and colleagues[24] suggest that higher empathy could strengthen social deterrents of suicidal ideation, thereby providing some support for this assertion. Further, a recent systematic review of reviews concluded that both objective social isolation and subjective perceptions of loneliness are risk factors for suicidal ideation.[25]

This investigation sought to shed light on the role of relatively neglected determinants of suicidal ideation, to examine if they predict suicidal ideation over and above the effects of known risk-factors, such as mental health problems, multi-morbidity and economic adversity, with a view to informing suicidality policy, prevention and risk management practice.

METHODS

Participants and sampling procedure

A cross-sectional Household Health Survey (HHS) was conducted in the north west of England as part of the National Institute for Health Research Collaboration for Leadership in Applied Health Research and Care – North West Coast (NIHR CLAHRC-NWC). A random area probability sampling strategy was adopted. Twenty high deprivation neighbourhoods and eight less deprived neighbourhoods were selected, and random addresses were contacted within those neighbourhoods. The areas were selected in consultation with local authority representatives based on the following considerations: population size (5,000-10,000 people), level of disadvantage (as measured via Index of Multiple Deprivation), coherent shared identity, and available infrastructure for policy delivery. Overall, 4319 people were recruited

For peer review only - http://bmjopen.bmj.com/site/about/guidelines.xhtml

between August 2015 and January 2016. There were 809 participants from low deprivation neighbourhoods and 3510 from high deprivation neighbourhoods. This was consistent with the sampling strategy, which had higher targets for high deprivation areas due to the overall project focus of health inequalities. The sample comprised 1854 (43%) men and 2465 (57%) women with ages ranging from 18 to 95 years (M = 49.12, SD = 19.13). Consistent with the demographic composition of the region,[26] most participants (89%) indicated that they were from White European ethnic backgrounds. Participants were reimbursed with a £10 voucher in return for their participation. The adjusted response rate was 61%. A more detailed description of the sampling method and neighbourhood selection procedures can be found in Giebel et al.[26]

The research was approved by University of Liverpool Committee on Research Ethics (Ref: RETH00836 and IPHS-1516-SMC-192) and conforms to the principles embodied in the Declaration of Helsinki. Written informed consent was obtained from all participants.

Patient and Public Involvement

Five Public Advisors (PAs) from the National Institute for Health Research Applied Research Collaboration North West Coast (NIHR ARC NWC) were recruited as investigators based upon their personal interest and/or experience of suicide and/or self-harm. PAs had an equal voice within the project team, which also comprised academics, researchers and clinicians. One PA agreed to be a named co-author within the project dissemination materials, whilst the remaining four PAs declined named co-authorship. PAs helped to shape the research question, key objectives and variables of interest and contributed to the choice of statistical models used. They reviewed the paper commenting on accuracy and ensured the wording was accessible to the public. The PA co-author was asked to prepare a plain English summary of the paper for inclusion on a University website accessible to the public. This advisor has also agreed to be available for wider dissemination of the study results at conferences and with local interest groups as agreed with research personnel.

For peer review only - http://bmjopen.bmj.com/site/about/guidelines.xhtml

Measures

A subset of the overall HHS questions was included in the analysis reported here. Decisions about which variables to include were informed by current suicidal ideation theories and research evidence, as well as by extensive consultation with members of the project team, including clinicians, academics, and people with lived experience. All variables were derived from single or multiple items of existing instruments recoded where necessary to between 2 and 5 categories for analysis. Coding and sources for all study measures are provided in Supplementary File Table 1. Information about **Suicidal ideation** was derived from response to item 9 in the Patient Health Questionnaire (PHQ-9)[27] which elicits the frequency of "thoughts that you would be better off dead or of hurting yourself" in the preceding two weeks. Responses of 'several days' or higher frequency were coded as '1' and 'not at all' as '0'. **Socio-demographic variables and caring responsibilities** were coded in accordance with UK Office for National Statistics national census categories.[28] Other variables were measured as follows: **housing quality**: English Housing Survey[29], 3 items; **financial situation**: Wealth and Assets Survey[30] ,1 item; **physical health status**: EQ-5D[31] , 5 items; **social capital and neighbourhood belonging**: Community Life Survey[32] , 3 items; **physical health conditions**: Adult Psychiatric Morbidity Study[33], 23 conditions, 1 item each; **medication side effects**: Health Survey for England[34], 2 items; **alcohol consumption and smoking**: Merseyside Lifestyle Survey[35], 1 item each; **depression**: PHQ-9 [27], 8 items as item 9 (suicidal ideation) was used as the dependent variable. ; **anxiety**: Generalised Anxiety Disorder Questionnaire (GAD7)[36], sum of 7 items; **paranoia**: Five-item Persecution and Deservedness Scale (PaDS-5)[37], sum of 5 items; **wellbeing**: Warwick-Edinburgh Mental Well-being Scale (WEMWBS)[38], sum of 7 items, abbreviated); **self-esteem**: Self-Esteem Scale[39], 1 item; **empathy**: Interpersonal Reactivity Index (IRI)[40], sum of 5 items, abbreviated; **hopelessness**: sum of 2 items (*Brief-H-Pos:* reverse scored)[41]; and **locus of control**: Levenson Locus of Control Scale[42], sum of 9

For peer review only - http://bmjopen.bmj.com/site/about/guidelines.xhtml

items, abbreviated. Descriptive statistics for each measure are provided in Supplementary File Table 2.

Data analysis strategy and preliminary analyses

Data were analysed using Stata version 12.[43] As the dependent variable, suicidal ideation (SI), was highly skewed (*S-W* = .92, $p < .00001$), the variable was re-coded into 0 = *suicidal ideation absent*, 1 = *suicidal ideation present*. While dichotomization of variables results in potential reductions in effect sizes and power, as well as loss of information, it is recommended for instances of severely skewed data where many participants fall at the extreme end of a scale as is the case here.[44] Specifically, 89% (n = 3833) of the sample reported having no SI over the previous 2 weeks, while 454 participants reported having SI (every day: $n = 99$; more than half the days: $n = 138$, Several days: $n = 217$). Given the possibility of collinearity between the four mental health symptoms and between mental health symptoms and SI, Pearson's product moment and Pearson's point-biserial correlations were conducted to examine bivariate relationships. As shown in Table 1, all predictors were moderately correlated with the criterion. The strongest association was between depression and SI, r_{pb} (4285) = .57, $p < .001$. When examining collinearity between predictors, anxiety and depression were highly significantly positively correlated, r (4303) = .79, $p < .001$. As the correlation was below .8 and anxiety and depression represent distinct theoretical constructs, multicollinearity was not considered problematic for the logistic regression analysis.[45]

Two logistic regression (LR) analyses were conducted with SI regressed on the socioeconomic, health and lifestyle variables. Standard errors were adjusted to account for the clustered nature of the data using the *svyset* command and the 28 neighbourhoods as clusters. The data was also weight-adjusted to account for demographic variation in non-response. The models provided estimates of the odds ratio (OR) of SI associated with each variable, while holding all other variables in the model constant. Because the mental health symptoms explain

For peer review only - http://bmjopen.bmj.com/site/about/guidelines.xhtml

a substantial portion of variance in SI, we constructed models both excluding (Model 1) and including (Model 2) symptoms to quantify the association between social and health factors and SI, as well as their predictive power above and beyond the effects of mental health.

Analysis indicated that no variable was missing more than 5% of values and only one variable (housing quality) was missing more than 1% of values. A Little's MCAR test indicated data was not missing completely at random, $\chi^2(335) = 457.35$, $p < .001$. Follow-up Separate Variance t-tests with threshold set to 1% indicated that housing quality missingness was associated with the mental health indicators of depression, anxiety, paranoia and wellbeing (*p*s < .005). Because Little's MCAR is highly sensitive to large sample sizes and missingness was extremely low for all variables, listwise deletion was used to account for missing values in each analysis. This resulted in $n = 3966$ for model 1 and $n = 3940$ for model 2.

Table 1. *Bivariate correlations between mental health variables*

Variable	1	2	3	4	5
1. Suicidal ideation	-	.57***	.51***	.34***	-.34***
2. Depression	-	-	.79***	.50***	-.52***
3. Anxiety	-	-	-	.55***	-.52***
4. Paranoia	-	-	-	-	-.39***
5. Wellbeing	-	-	-	-	-

*** $p < .001$

For peer review only - http://bmjopen.bmj.com/site/about/guidelines.xhtml

RESULTS

Model 1: Logistic regression predicting suicidal ideation without adjusting for mental health variables

The overall model was significant, $F(62, 3877) = 6.42$, $p < .0001$. Significant effects with alpha set to .001, .01, and .05 are highlighted and adjusted odds ratios are reported alongside confidence intervals within Table 2 below, whilst both significant and non-significant effects for all variables are reported within Supplementary File Table 3. Age was a significant predictor of SI. All younger age groups reported significantly higher odds of SI compared to the base category of 65+ years. Eighteen to twenty-four-year olds had the highest increase in odds of SI relative to the base category. People from black and minority ethnic (BME) backgrounds had significantly higher odds of SI compared to people from white European backgrounds. Living in lower quality housing, being in the same financial position as 12 months ago and not currently being employed were all significantly associated with higher odds of SI. Experiencing moderate or extreme pain/discomfort increased the odds of SI. Having side effects from medication was associated with higher odds of SI. Of the physical health condition variables, having a stroke or a hearing condition in the previous 12 months was associated with significantly increased odds of SI. Reporting arthritis was associated with significantly lower odds of SI. Examination of the psychological risk-factors of mental illness revealed that higher levels of self-esteem were significantly associated with d lower odds of SI. Similarly, higher levels of empathy were associated with lower odds of experiencing SI. Conversely, higher levels reported on the external locus of control ‘chance’ subscale were significantly associated with higher odds of SI. Feeling hopeless was also associated with higher odds of SI.

For peer review only - http://bmjopen.bmj.com/site/about/guidelines.xhtml

Lifestyle factors were also significantly associated with SI. Being a current occasional or heavy smoker was associated with higher odds of SI. Abstaining from alcohol reduced odds of SI by 37% relative to drinking within the recommended limits.[46]

Of the social capital variables, neighbourhood belonging was the only significant predictor, whereby an increase in sense of belonging was associated with lower odds of SI.

Model 2: Logistic regression predicting suicidal ideation adjusting for mental health variables

The overall model was significant, $F(69, 3844) = 9.38$, $p < .0001$; however, the profile of significant risk factors was somewhat different compared with model 1, as reported in Table 2 below. After adjusting for mental health symptoms, identifying as lesbian, gay, bisexual, queer or transgender (LGBQT) or BME was associated with significantly higher odds of SI. Reporting being in the same financial position as in the previous 12 months was significantly associated with 2.29 times higher odds of SI compared to being in a worse position than 12 months ago. No other demographic or socioeconomic variables significantly predicted SI.

Reporting a cancer diagnosis was significantly associated with 3.90 higher odds of SI, while reporting arthritis was associated with reduced odds of SI.

Self-esteem, hopelessness, and locus of control were not associated with SI when mental health variables were taken into account. However, increased Empathy scores were associated with a reduction in odds of SI.

Past and present smoking behaviours were unrelated to SI in this model. Abstaining from alcohol was significantly associated with lower odds of SI. No social capital variables were associated with SI.

All of the mental health symptoms variables were associated with higher risk of SI. Specifically, anxiety and paranoia were associated with significantly higher odds of SI

For peer review only - http://bmjopen.bmj.com/site/about/guidelines.xhtml

while depression showed the strongest relationship with SI with each 1 unit increase on the PHQ-9 being associated with 7.25 higher odds of SI. Wellbeing was not related to SI.

Table 2. *Statistically significant logistic regression variables predicting suicidal ideation excluding (Model 1) and including (Model 2) mental health variables.*

	Model 1			*Model 2*	
Predictors	*Adjusted Odds Ratio of suicidal ideation*	*95% CI*		*Adjusted Odds Ratio of suicidal ideation*	*95% CI*
Mental health					
Depression	-	-		*7.24****	*5.22, 10.07*
Anxiety	-	-		*1.56***	*1.13, 2.17*
Paranoia	-	-		*1.36**	*1.72*
Demographics					
Age (65+)					
18-24	*5.50****	*2.74, 11.06*		*0.95*	*.38, 2.38*
25-44	*4.50****	*2.48, 8.15*		*1.62*	*.84, 3.15*
45-64	*2.82***	*1.68, 4.73*		*1.1*	*.60, 2.02*
Black & Minority Ethnic (BME)	*1.88**	*1.01, 3.49*		*1.93**	*1.04, 3.62*

For peer review only - http://bmjopen.bmj.com/site/about/guidelines.xhtml

LGBTQ	*1.93*	*.77, 4.83*	*2.73**	*1.00, 7.46*
Socioeconomic status				
Problems with housing	*1.67****	*1.26, 2.23*	*1.34*	*.95, 1.89*
Financial position (worse)				
Same	*1.68**	*1.02, 2.76*	*2.29***	*1.24, 4.23*
Non-employment	1.43*	1.00, 2.03		
Health problems (EQ-5D)				
Pain	*1.62**	*1.09, 2.40*	*0.98*	*.61, 1.56*
Side effects				
No medication				
Never bother	1.47*	1.02, 2.12		
Bother a little	2.93**	1.35, 6.36		
Bother somewhat	2.31**	1.23, 4.34		
Bother a lot	2.64**	1.37, 5.10		
Health conditions				
Cancer	*1.74*	*.80, 3.77*	*3.90***	*1.40, 10.84*

For peer review only - http://bmjopen.bmj.com/site/about/guidelines.xhtml

Ear	*2.02***	*1.20, 3.41*	*1.24*	*.59, 2.59*
Stroke	*2.01**	*1.06, 3.81*	*1.63*	*.57, 4.68*
Arthritis	*.59**	*.40, .88*	*.54**	*.30, .95*
Alcohol consumption				
None (0 units)	*.63***	*.46, .87*	*.61**	*.42, .90*
Smoking status				
Current occasional smoking	*1.99**	*1.04, 3.81*	*1.78*	*.80, 3.96*
Current daily smoking	*1.92****	*1.35, 2.74*	*1.51*	*.98, 2.33*
Psychological factors				
Empathy	*.82**	*.70, .96*	*.72***	*.59, .88*
Self-esteem	*.81****	*.75, .88*	*0.97*	*.87, 1.09*
Locus of control (chance)	*1.35***	*1.11, 1.64*	*1.23*	*.95, 1.60*
Social capital				
Neighbourhood Belonging	*.69**	*.48, .97*	*0.9*	*.58, 1.38*

* $p < .05$, ** $p < .01$, *** $p < .001$

For peer review only - http://bmjopen.bmj.com/site/about/guidelines.xhtml

DISCUSSION

Principal findings

This study was uniquely able to investigate wider determinants of suicidal ideation including demographic, socio-economic, housing and neighbourhood quality, mental health, physical health, wellbeing, lifestyle and social capital factors. Utilisation of a community-based population enhances generalisability of these findings beyond the clinical populations typically used within suicidology literature. In addition, the application of a shorter measurement timeframe (i.e. two-weeks) within suicide research is relatively new, but in line with clinical risk management practices.

Congruent with current suicide prevention literature[47] depression, anxiety and paranoia were all identified as risk factors for suicidal ideation. The strongest of these effects was related to depression insofar as each 1 unit increase on the PHQ-9 was associated with a seven-fold increase in odds of suicidal ideation.

Physical health conditions that are enduring and/or debilitating in nature, or life threatening, have been shown to correlate with suicidal ideation both dependently[48] and independently from common mental disorders[49] and our study supports these findings. Specifically, pain/discomfort, having cancer, a stroke or hearing problems, may engender perceptions of burdensomeness,[50] defeat and entrapment,[51] psychological and/or physiological pain and/or hopelessness,[52] which are all suggested pre-conditions for suicidal ideation. However, 'arthritis' was found to be a protective factor against suicidal ideation in this sample. A possible explanation could be that this is a common condition, particularly amongst older people and therefore individuals may feel less 'alone' living with arthritis and/or there may be less stigma and more formal/informal support for sufferers. Another potential explanation of this unexpected finding is that treatments for arthritic pain may have antidepressant effects.[53,54] This finding requires further investigation.

For peer review only - http://bmjopen.bmj.com/site/about/guidelines.xhtml

Perceptions of defeat and/or entrapment may also underpin findings from previous research which has shown that cancer and dementia patient family caregivers have higher levels of suicidal ideation with comorbid depression, whilst older age and having clear reasons for living reduce such risks.[55] However, carer status was not found to be a statistically significant predictor in this sample. Carer burden may be exacerbated by longer duration of carer role and particularly challenging patient needs. These aspects of caring were not examined within this survey and may explain the differences in findings.

Poor quality housing, being in the same financial position as the previous year and being unemployed were identified risk factors for suicidal ideation within this study, which reflect the known wider determinants of health inequalities[56] and may also represent perceptions of defeat/entrapment that underpin suicidal ideation. Without adjustment for mental health factors, younger age – particularly being aged between 18 and 24 - was found to increase suicidal ideation risk, which is again in line with previous literature.[8] Similarly, hopelessness and believing your life to be determined by 'chance' were also risk factors for suicidal ideation. These findings may reflect reduced objective or subjective personal agency and an opportunity for targeted educational, occupational and clinical interventions.

Our findings show that higher self-esteem is a protective factor against suicidal ideation, again corroborating previous research.[57, 58] Further, both theory and research suggest that higher levels of social capital have a positive impact upon mental health[59] and our analysis excluding mental health factors supports this.

Thwarted belonging relates to the fundamental need to belong and when this need is compromised it can underpin suicidal ideation and behaviour.[50] Indeed, Wasler et al[60] found that in a sample of people with first episode psychosis, perceived burdensome and thwarted belonging were elevated in people with recent suicidal ideation compared with individuals without recent suicidal ideation. Joiners'[50] theory may explain the increased risk of suicidal

For peer review only - http://bmjopen.bmj.com/site/about/guidelines.xhtml

ideation from minority status groups such as identifying as LGBQT or being from an ethnic minority background, as demonstrated within previous research[61] and by the results from this study. Further, as the sample was from a predominantly white British population, it is possible that social factors such as discrimination and social exclusion experienced by people from BME groups may have contributed to their greater SI vulnerability. In support of this, neighbourhood belonging was found to be a protective factor against suicidal ideation within this study, providing additional support to the argument that a sense of belonging can support better mental health. Importantly, neighbourhood belonging has been found to be more prominent in lower SES populations than in higher SES populations where wider social networks play a more prominent role in sense of belonging.[62]

Given the reported preference for self-management of suicidal ideation,[5] individuals may use lifestyle behaviours such as smoking and alcohol consumption as coping mechanisms. Our findings highlight smoking and higher levels of alcohol consumption as risk factors for suicidal ideation.

Our findings indicate that higher levels of empathy reduce the risk of SI which remain when adjusting for mental health factors. Zhang, and colleagues[24] suggestion that higher empathy strengthens social deterrents to suicidal ideation and behaviour, provides a possible explanation for this novel finding in a non-clinical sample. Having the automatic capacity to take the perspective of loved ones left behind when one is contemplating suicide would provide a strong, natural barrier to end such thoughts and to bar completion.

Limitations

Whilst based on validated measures with a large representative sample, the methodology adopted here has certain limitations which must be considered when interpreting the results. Firstly, the survey used in this study is entirely based on self-report methods. Reporting bias can be an issue, due to the sensitive nature of some of the interview questions.

For peer review only - http://bmjopen.bmj.com/site/about/guidelines.xhtml

Secondly, capturing suicidal ideation using a single item measure may have resulted in over-simplification, as the validity of single items when detached from a larger instrument can be contested. However, the large sample size may have mitigated these problems. Thirdly, again despite the large sample size, generalisability beyond the sample studied here must be exercised with caution. This was a regional rather than a national sample and there may be specific economic or cultural factors which do not apply beyond the North West of England. The demographic characteristics of the sample were restricted in terms of age and ethnicity. The exclusion of participants aged under 18 years prevents conclusions being drawn about the adolescent age group where suicidal ideation is common. Similarly, the aggregation of non-white ethnicities into a single BAME category in the analysis here eradicates any possible examination of differences within these non-White groups. Fourthly, the suicidal ideation outcome variable does not provide information on actual suicidal behaviour which is the key clinical need to be addressed. Finally, the suicidal ideation variable was also very skewed and the variable needed to be dichotomised to increase power and minimise error variance. However, this was at the expense of nuance in the findings insofar as our data cannot elucidate potential differences between higher and lower frequency suicidal ideation.

Clinical Implications

While being mindful of the limitations of this study, some of the novel findings reported, if replicated, have clear clinical implications. Perhaps the most important of these is consistent with public mental health approaches to intervention including social prescribing routes for prevention of distress and promotion of wellbeing. These approaches stress the importance of building community and sense of belonging. Our findings indicate that peer support groups for chronic health conditions such as cancer, stroke and hearing could mitigate thoughts of suicide by providing social support, a reason to continue and a source of relational wellbeing, as well as potentially adding to knowledge about one's condition and how to cope

For peer review only - http://bmjopen.bmj.com/site/about/guidelines.xhtml

with it. The same is true for particular groups shown in our analysis to be more prone to suicidal ideation, including LGBTQ and ethnic minority groups. Neighbourhood support groups could go some way to increase sense of belonging to community for minority groups. Relational approaches to support individual mental health have been advocated for some time and there is a wealth of robust evidence demonstrating the role of peer support in enhancing wellbeing in the context of mental and physical heath difficulties.[63-66] Our analysis shows that the benefits of these approaches may well extend to the prevention of suicidal ideation. Furthermore, it is likely that increased interpersonal contact with similar individuals in the context of support or neighbourhood groups may, in time, translate to enhanced perspective-taking skills which, in this sample, was found to be a psychological variable negatively associated with proneness to suicidal thoughts. Thus, communities of place, support and interest may provide solutions to the experience of suicidal ideation and may prevent such thoughts escalating to suicidal acts.

Conclusion

Identification of risk and protective factors for SI can support the implementation of tailored clinical and non-clinical interventions. This study has identified new risk and protective factors for suicidal ideation using a randomly selected large community-based sample from disadvantaged and less disadvantaged neighbourhoods. Using this approach, as well as statistically adjusting for demographic variation in non-responses, enhances the validity of the study, especially the generalisability of its findings beyond the clinical populations typically used within suicidality literature. This study suggests that it could be useful to increase community belonging and community support within a public health approach for vulnerable groups (e.g. those with cancer) and peer support for people who identify as LGBTQ and/or BME. Also increasing empathic functioning, potentially through involvement with support groups may be an effective strategy for reducing suicidal ideation.

COMPETING INTERESTS

For peer review only - http://bmjopen.bmj.com/site/about/guidelines.xhtml

Dr McIntyre reports grants from NIHR, during the conduct of the study. Remaining authors have nothing to disclose.

FUNDING

This study is part-funded by the NIHR CLAHRC NWC.

AUTHOR CONTRIBUTIONS

The authors have all substantially contributed to this paper.

- HM is the lead author who conceived the study, provided interpretation of the data for this paper and drafted all sections of the manuscript, with the exception of the methods and 'Clinical Implications' section.
- JMcI has contributed to the design of the study, conducted the data analysis, drafted the methods section and provided substantial critical comments to the whole manuscript at the drafting stage.
- AH has contributed to the design of the study and interpretation of the data, provided substantial critical comments at the drafting stage and conducted final manuscript editing and proofing.
- RW has contributed to the drafting of the methods section, interpretation of the data and provided critical comments at the drafting stage.
- TC has contributed to the design of the study, interpretation of the data, drafted the 'Public and Patient Involvement' section and provided critical comments at the drafting stage.
- RC has contributed to the design of the study, interpretation of the data, drafted the 'Clinical Implications' section and provided critical comments at the drafting stage.

All authors have approved the final version of the manuscript.

For peer review only - http://bmjopen.bmj.com/site/about/guidelines.xhtml

ACKNOWLEDGMENTS

We would like to acknowledge: Dr Cecil Kullu, Emma Mullin, Elizabeth Fuller, Paula Gross, Jane Shelton, Stuart Wood, and Farheen Yameen for contributing to the design of this project and its' governance.

TC is part funded / supported by The National Institute for Health Research Applied Research Collaboration North West Coast (NIHR ARC NWC). The views expressed are those of the authors and not necessarily those of the NIHR, NHS, or Department of Health and Social Care.

EXCLUSIVE LICENCE

I, the Submitting Author has the right to grant and does grant on behalf of all authors of the Work (as defined in the below author licence), an exclusive licence and/or a non-exclusive licence for contributions from authors who are: i) UK Crown employees; ii) where BMJ has agreed a CC-BY licence shall apply, and/or iii) in accordance with the terms applicable for US Federal Government officers or employees acting as part of their official duties; on a worldwide, perpetual, irrevocable, royalty-free basis to BMJ Publishing Group Ltd ("BMJ") its licensees and where the relevant Journal is co-owned by BMJ to the co-owners of the Journal, to publish the Work in Journal of Epidemiology & Community Health and any other BMJ products and to exploit all rights, as set out in our licence.

The Submitting Author accepts and understands that any supply made under these terms is made by BMJ to the Submitting Author unless you are acting as an employee on behalf of your employer or a postgraduate student of an affiliated institution which is paying any applicable article publishing charge ("APC") for Open Access articles. Where the Submitting Author wishes to make the Work available on an Open Access basis (and intends to pay the

For peer review only - http://bmjopen.bmj.com/site/about/guidelines.xhtml

relevant APC), the terms of reuse of such Open Access shall be governed by a Creative Commons licence – details of these licences and which Creative Commons licence will apply to this Work are set out in our licence referred to above.

DATA SHARING STATEMENT

The dataset from the North West Coast Household Health Survey will be made publicly available after an embargo period.

REFERENCES

1. World Health Organisation (WHO). *Suicide. Key Facts*. https://www.who.int/news-room/fact-sheets/detail/suicide, 2019.
2. Department of Health (DoH). *No Health without Mental Health: a cross-government outcomes strategy*. https://www.gov.uk/government/publications/no-health-without-mental-health-a-cross-government-outcomes-strategy, 2011.
3. Nock, M. K., Borges, G., Bromet, E. J., Alonso, J., Angermeyer, M., Beautrais, A., Bruffaerts, R., Chiu, W. T., de Girolamo, G., Gluzman, S., de Graaf, R., Gureje, O., Haro, J. M., Huang, Y., Karam, E., Kessler, R. C., Lepine, J. P., Levinson, D., Medina-Mora, M. E., Ono, Y., … Williams, D. Cross-national prevalence and risk factors for suicidal ideation, plans and attempts. *The British journal of psychiatry: the journal of mental science*, 2008; *192*(2), 98–105. https://doi.org/10.1192/bjp.bp.107.040113.
4. van Spijker, BAJ; van Straten, A; Kerkhof, AJFM; Hoeymans, N; Smit, F. Disability weights for suicidal thoughts and non-fatal suicide attempts. *Journal of affective disorders*, ISSN: 1573-2517, 2011, Vol: 134, Issue: 1-3, Page: 341-7. DOI10.1016/j.jad.2011.05.020.
5. Bruffaerts, R., Demyttenaere, K., Hwang, I., Chiu, W. T., Sampson, N., Kessler, R. C., Alonso, J., Borges, G., de Girolamo, G., de Graaf, R., Florescu, S., Gureje, O., Hu, C., Karam, E. G., Kawakami, N., Kostyuchenko, S., Kovess-Masfety, V., Lee, S., Levinson, D., Matschinger, H., … Nock, M. K. Treatment of suicidal people around the world. *The British journal of psychiatry : the journal of mental science*, 2011; *199*(1), 64–70. https://doi.org/10.1192/bjp.bp.110.084129.
6. Alonso J, Angermeyer MC, Bernert S, Bruffaerts R, Brugha TS, Bryson H, de Girolamo G, Graaf R, Demyttenaere K, Gasquet I, Haro JM, Katz SJ, Kessler RC, Kovess V, Lépine JP, Ormel J, Polidori G, Russo LJ, Vilagut G, Almansa J, Arbabzadeh-Bouchez S, Autonell J, Bernal M, Buist-Bouwman MA, Codony M, Domingo-Salvany A, Ferrer M, Joo SS, Martínez-Alonso M, Matschinger H, Mazzi F, Morgan Z, Morosini P, Palacín C, Romera B, Taub N, Vollebergh WA, ESEMeD/MHEDEA 2000 Investigators, European Study of the Epidemiology of Mental Disorders (ESEMeD) Project. *Acta Psychiatr Scand* Suppl. 2004; (420):8-20.
7. McManus S, Bebbington P, Jenkins R, Brugha T. (eds.) *Mental health and wellbeing in England: Adult Psychiatric Morbidity Survey 2014*. Leeds: NHS Digital, 2016.
8. Borges G, Nock MK, Haro Abad JM, et al. Twelve-Month Prevalence of and Risk Factors for Suicide Attempts in the World Health Organization World Mental Health Surveys. *Journal of Clinical Psychiatry* 2010:1617. doi: 10.4088/jcp.08m04967blu.

For peer review only - http://bmjopen.bmj.com/site/about/guidelines.xhtml

9. McClatchey, K., Murray, J., Rowat, A., & Chouliara, Z. Risk Factors for Suicide and Suicidal Behavior Relevant to Emergency Health Care Settings: A Systematic Review of Post-2007 Reviews. *Suicide & Life-Threatening Behavior*, 2017; *47*(6), 729–745.
10. O'Connor, R.C., Kirtley, O.J. The Integrated Motivational-Volitional Model of Suicidal Behaviour. *Philosophical Transactions of the Royal Society B. 2018;* 373: 20170268. http://dx.doi.org/10.1098/rstb.2017.0268.
11. Klonsky E.D. & May, A.M. The Three-Step Theory (3ST): A New Theory of Suicide Rooted in the "Ideation-to-Action" Framework. *International Journal of Cognitive Therapy*, 2015; 8(2), 114–129.
12. Baumeister, R.F. Suicide as escape from self. *Psychological Review,* 1990; *97*, 90-113.
13. Linehan, M.M. *Cognitive-Behavioral treatment of borderline personality disorder*. New York: Guilford Press, 1993.
14. Brown, M.Z. Linehan's theory of suicidal behavior: Theory, research, and dialectical behavior therapy. In: T. Ellis (Ed.), *Cognition and Suicide: Theory, Research, and Practice*. American Psychological Association, 2006.
15. Marmot, Michael (2005) Social determinants of health inequalities. *Lancet*, 365 (9464). pp. 1099-104. ISSN 1474-547X
16. Handley, T. E., Inder, K. J., Kay-Lambkin, F. J., Stain, H. J., Fitzgerald, M., Lewin, T. J., Attia, J. R., & Kelly, B. J. Contributors to suicidality in rural communities: beyond the effects of depression. *BMC Psychiatry*, 2012; *12*, 105.
17. Barnes, M., Caullinane, C., Scott, S. & Silvester, H. *People Living in Bad Housing: Numbers and Health Impacts*. London: National Centre for Social Research, 2013.
18. Rosengard, C.; Folkman, S. Suicidal ideation, bereavement, HIV serostatus and psychosocial variables in partners of men with AIDS. *AIDS Care* 1997, 9, 373–384.
19. O'Dwyer ST, Moyle W, Pachana NA, Sung B, Barrett S. Feeling that life is not worth living (death thoughts) among middle-aged, Australian women providing unpaid care. *Maturitas*. 2014 Apr;77(4):375-9. doi: 10.1016/j.maturitas.2014.01.013. Epub 2014 Feb 7. PMID: 24602554.
20. Anderson, J.G.; Eppes, A., & O'Dwyer,S.T. "Like Death is Near": Expressions of Suicidal and Homicidal Ideation in the Blog Posts of Family Caregivers of People with Dementia. *Behavioral Sciences*, 2019; *9*(3), 22.
21. Park, B., Kim, S. Y., Shin, J.-Y., Sanson-Fisher, R. W., Shin, D. W., Cho, J., & Park, J. H. Suicidal Ideation and Suicide Attempts in Anxious or Depressed Family Caregivers of Patients with Cancer: A Nationwide Survey in Korea. *PLoS ONE*, 2013; 8(4), 1–7.
22. Cristancho, P., O'Connor, B., Lenze, E. J., Blumberger, D. M., Reynolds, C. F., 3rd, Dixon, D., & Mulsant, B. H. Treatment Emergent Suicidal Ideation in depressed older adults. *International journal of geriatric psychiatry*, 2017; *32*(6), 596–604. https://doi.org/10.1002/gps.4498.
23. Hamm BS, Rosenthal LJ. Psychiatric Aspects of Chloroquine and Hydroxychloroquine Treatment in the Wake of Coronavirus Disease-2019: Psychopharmacological Interactions and Neuropsychiatric Sequelae. *Psychosomatics*. 2020 Jul 8:S0033-3182(20)30209-7. doi: 10.1016/j.psym.2020.06.022. Epub ahead of print. PMID: 32800347; PMCID: PMC7341047.
24. Zhang K, Szanto K, Clark L, et al. Behavioral empathy failures and suicidal behavior. *Behaviour Research and Therapy* 2018 doi: 10.1016/j.brat.2018.10.019.
25. Calati, R., Ferrari, C., Brittner, M., Oasi, O., Olié, E., Carvalho, A. F., & Courtet, P. Review article: Suicidal thoughts and behaviors and social isolation: A narrative review of the literature. Journal of Affective Disorders, 2019; 245, 653–667.

For peer review only - http://bmjopen.bmj.com/site/about/guidelines.xhtml

26. Giebel, C., McIntyre, J.C., Alfirevic, A. *et al.* The longitudinal NIHR ARC North West Coast Household Health Survey: exploring health inequalities in disadvantaged communities. *BMC Public Health;* 2020; **20,** 1257. https://doi.org/10.1186/s12889-020-09346-5.
27. Kroenke K, Spitzer RL. The PHQ-9: *A New Depression Diagnostic and Severity Measure The nine-item Patient Health Questionnaire depression scale is a dual-purpose instrument that can establish provisional depressive disorder diagnoses as well as grade depression severity*. United States: SLACK INCORPORATED, 2002:509.
28. Office for National Statistics (ONS) *Classifications and harmonisation.* https://www.ons.gov.uk/methodology/classificationsandstandards, 2016.
29. UK Government. Ministry of Housing, Communities & Local Government. *English Housing Survey 2013 to 2014: questionnaire and physical survey form.* https://www.gov.uk/government/publications/english-housing-survey-2013-to-2014-questionnaire-and-physical-survey-form, 2015.
30. Office for National Statistics (ONS). *Wealth and Assets Survey QMI.* https://www.ons.gov.uk/peoplepopulationandcommunity/personalandhouseholdfinances/debt/methodologies/wealthandassetssurveyqmi, 2019.
31. Gusi N, Olivares P, Rajendram R. The EQ-5D health-related quality of life questionnaire. *Handbook of disease burdens and quality of life measures* 2010:87-99.
32. United Kingdom Cabinet Office. *Community Life Survey 2014-15*. https://www.gov.uk/government/publications/community-life-survey-2014-to-2015-statistical-analysis, 2015.
33. McManus S, Bebbington P, Jenkins R, et al. *Adult psychiatric morbidity survey: survey of mental health and wellbeing, England, 2014*: NHS Digital Leeds, UK, 2016.
34. National Health Service (NHS) Digital. *Health Survey for England – 2013*. https://digital.nhs.uk/data-and-information/publications/statistical/health-survey-for-england/health-survey-for-england-2013#summary , 2014.
35. Knowsley Council. NHS *Merseyside Lifestyle Survey*. http://www.knowsley.gov.uk/pdf/knowsley-health-and-lifestyle-survey-2012-13.pdf, 2013.
36. Spitzer RL, Kroenke K, Williams JBW, et al. A brief measure for assessing generalized anxiety disorder: The GAD-7. *Archives of Internal Medicine* 2006;166(10):1092-97. doi: 10.1001/archinte.166.10.1092.
37. Melo S, Corcoran R, Shryane N, et al. The persecution and deservedness scale. *Psychology & Psychotherapy: Theory, Research & Practice* 2009;82(3):247-60. doi: 10.1348/147608308X398337.
38. Tennant R, Hiller L, Fishwick R, et al. The Warwick-Edinburgh Mental Well-being Scale (WEMWBS): development and UK validation. *Health And Quality Of Life Outcomes* 2007;5:63-63.
39. Robins RW, Hendin HM, Trzesniewski KH. Measuring Global Self-Esteem: Construct Validation of a Single-Item Measure and the Rosenberg Self-Esteem Scale. *Personality & Social Psychology Bulletin* 2001(2).
40. Davis, M. H. A multidimensional approach to individual differences in empathy.*Catalog of Selected Documents in Psychology*, 1980; 10, 85.
41. Everson SA, Goldberg DE, Kaplan GA, et al. Hopelessness and risk of mortality and incidence of myocardial infarction and cancer. *Psychosom Med* 1996;58:113–21.
42. Levenson H. Multidimensional locus of control in psychiatric patients. *Journal of Consulting and Clinical Psychology* 1973;41(3):397-404. doi: 10.1037/h0035357.
43. Stata Statistical Software: Release 12. College Station, TX: StataCorp LP., 2011.

For peer review only - http://bmjopen.bmj.com/site/about/guidelines.xhtml

44. MacCallum RC, Zhang S, Preacher KJ, et al. On the practice of dichotomization of quantitative variables. *Psychological Methods* 2002;7(1):19-40. doi: 10.1037/1082-989X.7.1.19.
45. Midi H, Sarkar S, Rana S. Collinearity diagnostics of binary logistic regression model. *Journal of Interdisciplinary Mathematics* 2013;13:253-67. doi: 10.1080/09720502.2010.10700699.
46. Department of Health. UK Chief Medical Officers' low risk drinking guidelines: Department of Health London, 2016.
47. Hawton K, Casañas I Comabella C, Haw C, et al. Risk factors for suicide in individuals with depression: A systematic review. *Journal of Affective Disorders* 2013;147(1-3):17-28. doi: 10.1016/j.jad.2013.01.004
48. Kavalidou K, Smith DJ, O'Connor RC. The role of physical and mental health multimorbidity in suicidal ideation. *Journal of Affective Disorders,* 2017;209:80-85. doi: 10.1016/j.jad.2016.11.026
49. Qin P, Webb R, Kapur N, et al. Hospitalization for physical illness and risk of subsequent suicide: A population study. *Journal of Internal Medicine,* 2013;273(1):48-58. doi: 10.1111/j.1365-2796.2012.02572.x
50. Joiner TE. *Why people die by suicide*. Cambridge, MA: Harvard University Press 2005.
51. O'Connor R, Pirkis J. *The international handbook of suicide prevention*. Second edition. ed: Wiley, 2016.
52. Klonsky ED, May AM. Differentiating suicide attempters from suicide ideators: a critical frontier for suicidology research. *Suicide & Life-Threatening Behavior*, 2014;44(1):1-5. doi: 10.1111/sltb.12068.
53. Kappelmann N, Lewis G, Dantzer R, Jones PB, Khandaker GM. Antidepressant activity of anti-cytokine treatment: a systematic review and meta-analysis of clinical trials of chronic inflammatory conditions. *Mol Psychiatry*. 2018;23(2):335-343. doi:10.1038/mp.2016.167.
54. Citrome, L. and Weiss-Citrome, A. (2012), Antidepressants and the relief of osteoarthritic pain – Findings from a study examining adjunctive duloxetine. *International Journal of Clinical Practice,* 66: 431-433. doi:10.1111/j.1742-1241.2012.02899.x
55. O'Dwyer ST, Moyle W, Zimmer-Gembeck M, et al. Suicidal ideation in family carers of people with dementia. *Aging and Mental Health*, 2015:9p. doi: 10.1080/13607863.2015.1063109.
56. Public Health England (PHE) *Health profile for England: 2019.* https://www.gov.uk/government/publications/health-profile-for-england-2019, 2019.
57. Kotrbová K, Dóci I, Hamplová L, et al. Factors Influencing Suicidal Tendencies of Patients with Diagnosis of Attempted Suicide in Medical History and Potential Prevention of Relapse Prevention. *Central European Journal Of Public Health* 2017;25(4):271-76. doi: 10.21101/cejph.a4677
58. Eades A, Segal DL, Coolidge FL. Suicide Risk Factors Among Older Adults: Exploring Thwarted Belongingness and Perceived Burdensomeness in Relation to Personality and Self-Esteem. *International Journal of Aging and Human Development* 2019;88(2):150-67. doi: 10.1177/0091415018757214.
59. Putnam RD. *Bowling alone: the collapse and revival of American community*: Simon & Schuster 2000.
60. Wastler, H. M., Moe, A. M., Pine, J. G., & Breitborde, N. J. K. Perceived burdensomeness, thwarted belongingness and suicidal ideation among individuals with first-episode psychosis. *Early Intervention in Psychiatry*. 2020. doi: 10.1111/eip.13023.

For peer review only - http://bmjopen.bmj.com/site/about/guidelines.xhtml

61. Yıldız E. Suicide in sexual minority populations: A systematic review of evidence-based studies. *Archives of Psychiatric Nursing* 2018;32(4):650-59. doi: 10.1016/j.apnu.2018.03.003.
62. Robertson, D., Smyth, J. & McIntosh, I. *Neighbourhood identity: effects of time, location and social class.* Joseph Rowntree Foundation. https://www.jrf.org.uk/report/neighbourhood-identity-effects-time-location-and-social-class, 2008.
63. Fortuna, K. L., Naslund, J. A., LaCroix, J. M., Bianco, C. L., Brooks, J. M., Zisman-Ilani, Y., Muralidharan, A., & Deegan, P. Digital Peer Support Mental Health Interventions for People With a Lived Experience of a Serious Mental Illness: Systematic Review. *JMIR Mental Health*, 2020; *7*(4), e16460.
64. Stubbs B, Williams J, Shannon J, Gaughran F, Craig T. Peer support interventions seeking to improve physical health and lifestyle behaviours among people with serious mental illness: A systematic review. *Int J Mental Health Nurs*, 2016; 25: 484–495. doi:10.1111/inm.12256
65. Pinto BM, Stein K, Dunsiger S. Peers promoting physical activity among breast cancer survivors: a randomized controlled trial. *Health Psychol*. 2015;34(5):463–472. doi: 10.1037/hea0000120.
66. Pinto B, Stein K, Dunsiger S. Peer mentorship to promote physical activity among cancer survivors: effects on quality of life. *Psychooncology*. 2015;24(10):1295–1302. doi: 10.1002/pon.3884.

For peer review only - http://bmjopen.bmj.com/site/about/guidelines.xhtml

Supplementary File

Study measures - description

Suicidal ideation (SI)

Suicidal ideation was assessed utilising item 9 of the Patient and Health Questionnaire (PHQ-9).[27] Participants indicated how often over the last two weeks they had been bothered by “thoughts that you would be better off dead, or of hurting yourself in some way”. Response options ranged from 1 = *not at all* to 4 = *every day*.

Socio-economic demographics

Participants completed demographic measures, which included age, sex, ethnicity, sexuality, and relationship status. Measures of socioeconomic conditions included education level, employment status, change in financial circumstances over the past year, and housing quality.

Health

Physical health was assessed with the four physical health dimensions of the *EuroQuol five-dimension scale* (EQ-5D).[31] Health conditions were assessed with a single item from the *Adult Psychiatric Morbidity Survey: Survey of Mental Health and Wellbeing, England*[33] that allowed participants to indicate if they suffered from any of 22 named conditions, with the option to list an unnamed condition. Using this item, physical and mental health comorbidity was calculated and categorised into no conditions, mental health condition/s only, physical health condition/s only, and physical and mental health condition/s. Participants also indicated the extent to which medication side effects bothered them. Mental health was assessed using a series of validated instruments. Specifically, depression was measured using the nine-item PHQ-9[27] with item 9 (suicidal ideation) excluded as it was used as the dependent variable; anxiety was measured with the seven-item *Generalised Anxiety Disorder* scale (GAD-7),[36] paranoia was measured using the

For peer review only

persecution subscale of the *Persecution and Deservedness Scale* for symptoms of paranoia (PaDS-5),[37] and wellbeing was assessed with the *Warwick-Edinburgh Mental Wellbeing Scale* (WEMWBS).[38] Measures of psychological variables known to be associated with poor mental health were also obtained, including the *Single-Item Self Esteem Scale* (SISES),[39] along with abbreviated scales for empathy (Interpersonal Reactivity Index),[40] hopelessness (*Brief-H-Pos;* reverse scored)[41] and locus of control (Multidimensional Locus of Control Scales).[42]

Lifestyle factors

Alcohol consumption was measured using the number of units of alcohol participants reported consuming in the previous seven days. Participants were categorised in accordance with recommended drinking guidelines[66] as: abstaining, at or below recommended, above recommended, or more than double recommended levels. Past and present smoking behaviour was assessed with a single item adapted from the NHS Merseyside Lifestyle Survey.[35]

Social connectedness/capital

Participants indicated the number of hours they spent caring for a friend or family member. Social support, socialising, and neighbourhood belonging were assessed with single items from the *Community Life Survey*.[32]

For peer review only

Supplementary File Table 1. Coding and source of study measures

Variable	Source	Description/Example	Original scoring	Recoding for analysis
Age	Office for National Statistics	N/A	1 = Under 16 years<br>2 = 16-17 years<br>3 = 18-24 years<br>4 = 25-34 years<br>5 = 35-44 years<br>6 = 45-54 years<br>7 = 55-64 years<br>8 = 65-74 years<br>9 = 75+ years	1 = 18-24 years<br>2 = 25-44 years<br>3 = 45-64 years<br>4 = 65+ years

For peer review only

Sex	Office for National Statistics	N/A	1 = Male<br><br>2 = Female<br><br>3 = Other	1 = Male<br><br>2 = Female
Sexuality	Office for National Statistics	N/A	1 = Heterosexual or straight<br><br>2 = Gay or Lesbian<br><br>3 = Bisexual<br><br>4 = Other	0 = heterosexual<br><br>1 = LGBTQ
Ethnicity	Office for National Statistics	N/A	1 = English / Welsh / Scottish / Northern Irish / British<br><br>2 = Irish<br><br>3 = Gypsy or Irish Traveller<br><br>4 = Any other White background, *please specify*<br><br>5 = White and Black Caribbean	0 = White<br><br>1 = BME

For peer review only

			6 = White and Black African<br>7 = White and Asian<br>8 = Any other Mixed / Multiple ethnic background, *please specify*<br>9 = Indian<br>10 = Pakistani<br>11 = Bangladeshi<br>12 = Chinese<br>13 = Any other Asian background, *please specify*<br>14 = African<br>15 = Caribbean<br>16 = Any other Black / African / Caribbean background, *please specify*	

For peer review only

			17 = Arab<br><br>95 = Any other group, please specify	
Relationship status	Office for National Statistics	N/A	1 = Single/never married or in a same-sex civil partnership<br><br>2 = Married<br><br>3 = Separated, but still legally married<br><br>4 = Divorced<br><br>5 = Widowed<br><br>6 = Same-sex civil partnership<br><br>7 = Separated, but still in a same-sex civil partnership<br><br>8 = Formerly in a same-sex civil partnership that is now dissolved	0 = Partnered<br><br>1 = Single

For peer review only

			9 = Surviving partner from a same-sex civil partnership<br>10 = prefer not to say	
Non-employment	Office for National Statistics	N/A	1 = Going to school or college full time (including on vacation)<br>2 = In paid employment or self employed (or temporarily away)<br>3 = On a Government scheme for employment training<br>4 = Doing unpaid work for a business that you own, or that a relative owns<br>5 = Waiting to take up paid work already obtained<br>6 = Looking for paid work or a Government training scheme	0 = employed<br>1 = not employed

For peer review only

			7 = Intending to look for work but prevented by temporary sickness or injury<br>8 = Permanently unable to work because of long-term sickness or disability<br>9 = Retired from paid work<br>10 = Looking after the home or family<br>95 = Doing something else, specify	
Education	Office for National Statistics	Do you have any educational qualifications for which you received a certificate?<br><br>Do you have any professional, vocational or other work-related	1 = Yes, 2 = No<br><br>1 = Yes, 2 = No	1 = No qualifications<br>2 = Professional or vocational qualification<br>3 = Degree or higher

For peer review only

		qualifications for which you received a certificate?<br><br>What is your highest qualification?	 <br><br>1 = At degree level or above, 2 = Another kind of qualification	
Caring responsibilities	UK Census	Do you look after, or give any help or support to family members, friends, neighbours or others because of either a long term physical or mental ill-health / disability or problems related to old age? Do not count	1 = No<br>2 = Yes, 1-19 hours/week<br>3 = Yes, 20-49 hours/week<br>4 = Yes, 50+ hours/week	N/A

For peer review only

		anything you do as part of your paid employment.		
Housing quality	English Housing Survey	During the winter months, does condensation form on the windows or walls of any room in your home apart from the bathrooms or toilets?<br><br>During the winter months, are there patches of mould or fungus in any room in your home, apart from bathrooms or toilets?	1 = Yes, 2 = No, 3 = Spontaneous: don't know<br><br>1 = Yes, 2 = No, 3 = Spontaneous: don't know	0 = No problems<br>1 = One problem<br>2 = Two problems<br>3 = Problems with all three issues

For peer review only

		During the cold winter weather, can you normally keep comfortably warm in your living room?	1 = Yes, 2 = No, 3 = Spontaneous: don't know	
Financial Situation	Wealth and Assets Survey	Would you say your household is better off or worse off financially than you were a year ago?	1 = Better off<br>2 = About the same<br>3 = Worse off<br>4 = Don't know	1 = Better off<br>2 = About the same<br>3 = Worse off
Physical Health status	EuroQual Five Dimensional	Standardised instrument that assesses problems with mobility, self-care, engagement in usual activities, and pain.	1 = No problems<br>2 = Some Problems/Moderate problems<br>3 = Extreme problems/Unable	0 = No problems<br>1 = Some/Severe problems

For peer review only

	Scale (EQ-5D)			
Physical health conditions	Psychiatric Morbidity Survey	Have you ever had any of [these health conditions] over the past 12 months?	1 = Cancer<br>2 = Diabetes<br>3 = Epilepsy/fits<br>4 = Migraine or other frequent headaches<br>5 = Dementia or Alzheimer's disease<br>6 = Any mental health issue<br>7 = Cataracts / eyesight problems (even if corrected with glasses or contacts)<br>8 = Ear/hearing problems (even if corrected with a hearing aid)<br>9 = Stroke	For each condition:<br>0 = condition absent<br>1 = condition present<br>For physical and mental health comorbidity:<br>0 = No conditions

For peer review only

			10 = Heart attack/angina<br>11 = High blood pressure<br>12 = Bronchitis/emphysema<br>13 = Asthma<br>14 = Allergies<br>15 = Stomach ulcer or other digestive problems<br>16 = Liver problems<br>17 = Bowel/colon problems<br>18 = Bladder problems/incontinences<br>19 = Arthritis<br>20 = Bone, back joint or muscle problems<br>21 = Gout	1 = Mental health condition(s) only<br>2 = Physical health conditions(s) only<br>3 = Physical and mental health condition

For peer review only

			22 = Skin problems<br><br>95 = Any other condition	
Side effects	Health Survey for England (HSE)	Do any of your medications cause side effects or bother you in any way?<br><br>If yes, how much does it bothers you?	1 = Yes<br><br>2 = No<br><br>1 = A lot<br><br>2 = Somewhat<br><br>3 = A little<br><br>4 = Never	0 = No medication<br><br>1 = Never bothers<br><br>2 = Bothers a little<br><br>3 = Bothers somewhat<br><br>4 = Bothers a lot
Depression	Patient Health	Assesses how often participants had been bothered by pro problems such as "Feeling	1 = Not at all<br><br>2 = Several days	N/A

For peer review only

	Questionnaire (PHQ-9)	down, depressed, or hopeless" over the past two weeks.	3 = More than half the days<br>4 = Nearly every day	
Anxiety	Generalised Anxiety Disorder Questionnaire (GAD-7)	Assesses frequency of events such as "Being so restless that it is hard to sit still" over the past two weeks.	1 = Not at all<br>2 = Several days<br>3 = More than half the days<br>4 = Nearly every day	N/A
Paranoia	Five-item Persecution and Deservedness Scale (PaDS-5)	Assesses the extent to which people are suspicious of others' intentions. Participants rate their level of agreement with statements such as "I'm often suspicious of other people's intentions towards me."	1 = Strongly disagree<br>2 = Disagree<br>3 = Neither agree or disagree<br>4 = Agree<br>5 = Strongly agree	N/A

For peer review only

Wellbeing	The Warwick-Edinburgh Mental Well-being Scale (WEMWBS)	Abbreviated 7-item scale. Assesses general mood and wellbeing over the previous 2 weeks. Example statements include "I've been feeling relaxed" and "I've been thinking clearly".	1 = None of the time<br>2 = Rarely<br>3 = Some of the time<br>4 = Often<br>5 = All of the time	N/A
Self-esteem	Single-item Self-esteem Scale (SISE)	Validated scale assessing general levels of self-esteem. Participants rate their level of agreement on a 7-point scale with the statement "I have high self-esteem".	1 = Not very true of me<br>7 = Very true of me	

For peer review only

Empathy	Interpersonal Reactivity Index (IRI)	Abbreviated five-item version. Participants rate their level of agreement with statements such as "I am good at predicting how someone will feel".	1 = Strongly disagree<br>2 = Disagree<br>3 = Neither agree or disagree<br>4 = Agree<br>5 = Strongly agree	
Locus of control	Levenson Locus of Control Scale	Abbreviated nine-item version. Participants rated their level of agreement with statements such as "My life is determined by my own actions".	1 = Strongly disagree<br>2 = Disagree<br>3 = Neither agree or disagree<br>4 = Agree<br>5 = Strongly agree	N/A
Hopelessness	Brief-H-Pos	Participants rated their level of agreement with two statements: "The future seems to me to be	1 = Strongly disagree<br>2 = Disagree	1 = Strongly agree<br>2 = Agree

For peer review only

		hopeful and I believe that things are changing for the better" and "I feel that it is possible to reach the goals I would like to strive for"	3 = Neither agree or disagree<br>4 = Agree<br>5 = Strongly agree	3 = Neither agree or disagree<br>4 = Disagree<br>5 = Strongly disagree
Alcohol	Merseyside Lifestyle Survey	Participants indicated if they ever drank alcohol and if so how many of the following drinks they had consumed over the past seven days: pints of beer (low, normal and strong), pints of cider, bottles of alcopops, glasses of spirits, glasses of wine (small and large), glasses of fortified wine. These numbers were converted to	1 = none, moderate (<14 units/week),<br>2 = heavy (14-28 units/week),<br>3 = very heavy (>28 units/week).	N/A

For peer review only

		alcoholic units and then people were categorized into four levels of alcohol consumption based on the recommended usage of less than 14 units per week (Department of Health, 2016)		
Smoking status	Merseyside Lifestyle Survey	N/A	1 = Never smoked<br>2 = Past occasional smoker<br>3 = Past daily smoker<br>4 = Current occasional smoker<br>5 = Current daily smoker	N/A
Social capital	Community Life Survey	Assesses the extent to which participants agree they receive	1 = Definitely agree	0 = Disagree

For peer review only

		practical support ("If I needed help, there are people who would be there for me") and *social contact* ("If I wanted company or to socialise, there are people I can call on")	2 = Tend to agree<br>3 = Tend to disagree<br>4 = Definitely disagree<br>5 = Don't know	1 = Agree
Neighbourhood belonging	Community Life Survey	Participants were asked "how strongly you feel you belong to your immediate neighbourhood".	1 = Very strongly<br>2 = Fairly strongly<br>3 = Not very strongly<br>4 = Not at all strongly<br>5 = Don't know	0 = Not very/at all strongly<br>1 = Fairly/very strongly

For peer review only

Supplementary File Table 2. *Descriptive statistics for all variables. Discrepancies between total values and crosstabulated values due to non-response on the suicidal ideation variable*

	N (%)	*M* (*SD*)	*N(%) No suicidal ideation*	*N(% Suicidal ideation*
**Suicidal ideation in previous 2 weeks**				
Suicidal ideation	454 (10.6)	-	-	-
No suicidal ideation	3833 (89.4)	-	-	-
**Mental health symptoms**				
Depression (PHQ-9)	-	4.65(5.77)	-	-
Anxiety (GAD-7)	-	9.00 (4.33)	-	-
Paranoia (PaDS-5)	-	9.68 (4.38)	-	-
**Age**				
18-24 years	421 (9.8)	-	368(87.6)	52 (12.4)
25-44 years	1438 (33.3)	-	1247 (87.3)	181 (12.7)
45-64 years	1329 (30.8)	-	1162 (87.9)	160 (12.1)
65+ years	1129 (26.2)	-	1055 (94.6)	60 (5.4)
**Gender**				
Female	2465 (57.1)	-	2205 (90.2)	249 (9.8)
Male	1854 (42.9)	-	1628 (88.4)	214 (11.6)
**Ethnicity**				
Black and Minority Ethnic	455 (10.6)	-	414 (91.6)	38 (8.4)
White	3855 (89.4)	-	3411 (89.2)	415 (10.9)
**Sexuality**				
LGBTQ	65 (1.5)	-	50 (76.9)	15 (23.08)

For peer review only

Not LGBTQ	4246 (98.5)	-	3775 (89.6)	439 (10.4)
**Relationship status**				
Not married/civil partnership	2559 (49.4)	-	2209 (87.0)	330 (13.0)
Married/civil partnership	1747 (40.6)	-	1613 (92.9)	123 (7.1)
**Education**				
No qualifications	1718 (39.9)	-	1498 (88.0)	205 (12.0)
Professional/vocational certificate	1931 (44.8)	-	1727 (90.0)	192 (10.0)
Degree or higher	659 (15.3)	-	599 (91.3)	57 (8.7)
**Employment status**				
Employed	1745 (40.4)	-	1608 (92.5)	130 (7.5)
Not employed	2570 (59.6)	-	2233 (87.3)	323 (12.7)
**Housing quality (0-1 scale)**	-	.32 (.47)	-	-
-				
**Financial position compared with 12 months ago**				
Worse	679 (15.7)	-	549 (81.7)	123 (18.3)
Same	3090 (72.2)	-	2781 (90.6)	289 (9.4)
Better	510 (11.8)	-	471 (92.7)	37 (7.3)
**Caring responsibilities**				
None	3716 (86.0)	-	3298 (89.5)	389 (10.5)
1-19 hours/week	275 (6.4)	-	250 (91.6)	23 (8.4)
20-49 hours/week	100 (2.3)	-	86 (86.0)	14 (14.0)
50+ hours/week	228 (5.3)	-	199 (87.7)	28 (12.3)
**Health problems (EQ-5D; 0 – 1 scale)**				

For peer review only

Mobility	-	.24 (.43)	-	-
Self-care	-	.10 (.29)	-	-
Usual activities	-	.22 (.42)	-	-
Pain	-	.36 (.48)	-	-
**Health conditions**				
Cancer	120 (2.8)	-	99 (83.4)	20 (16.8)
Diabetes	341 (7.9)	-	299 (87.7)	42 (12.3)
Epilepsy	80 (1.9)	-	52 (65.8)	27 (34.2)
Migraine	348 (8.1)	-	270 (78.5)	74 (21.5)
Dementia	21 (0.5)	-	17 (81.0)	4 (19.0)
Eye	418 (9.7)	-	351 (85.2)	61 (14.8)
Ear	247 (5.7)	-	206 (85.1)	36 (14.9)
Stroke	97 (2.3)	-	76 (78.4)	21 (21.6)
Heart	280 (6.5)	-	240 (87.0)	36 (13.0)
Blood pressure	711 (16.5)	-	618 (88.0)	84 (12.0)
Bronchitis/Emphysema	113 (2.6)	-	91 (81.3)	21 (18.7)
Asthma	418 (9.7)	-	356 (85.2)	62 (14.8)
Allergies	185 (4.3)	-	158 (86.3)	25 (13.7)
Stomach/digestive	224 (5.2)	-	185 (83.0)	38 (17.0)
Liver	73 (1.7)	-	50 (70.4)	21 (29.6)
Bowel/colon	173 (4.0)	-	133 (77.8)	38 (22.2)
Bladder	136 (3.2)	-	108 (80.0)	27 (20.0)
Arthritis	728 (16.9)	-	634 (88.1)	86 (11.9)
Bone, back, joint, muscle	778 (18.0)	-	667 (86.4)	105 (13.6)
Gout	51 (1.2)	-	47 (92.2)	4 (7.8)
Skin	243 (5.6)	-	211 (87.6)	30 (12.4)
Other	267 (6.2)	-	235 (88.0)	32 (12.0)
**Side effects**				

For peer review only

No medication	2015 (46.8)	-	1842 (92.0)	160 (8.0)
Never bothers	1945 (45.2)	-	1732 (89.7)	198 (10.3)
Bothers a little	79 (1.8)	-	62 (78.5)	17 (21.5)
Bothers somewhat	145 (3.4)	-	106 (74.1)	37 (25.9)
Bothers a lot	125 (2.9)	-	85 (69.1)	38 (30.9)
**Alcohol consumption**				
Never (0 units/week)	1894 (44.0)	-	1674 (89.2)	202 (10.8)
Moderate (1-14 units/week)	1973 (45.8)	-	1761 (89.7)	202 (10.3)
High (14-28 units/week)	290 (6.7)	-	260 (90.0)	29 (10.0)
Very high (>28 units/week)	151 (3.5)	-	130 (87.3)	19 (12.7)
**Smoking status**				
Never	2107 (48.8)	-	1951 (93.3)	141 (6.7)
Past occasional smoking	286 (6.6)	-	257 (89.9)	29 (10.1)
Past daily smoking	671 (15.5)	-	605 (90.8)	61 (9.2)
Current occasional smoking	122 (2.8)	-	97 (79.5)	25 (20.5)
Current daily smoking	1118 (25.9)	-	910 (82.2)	197 (17.8)
**Psychological factors**				
Empathy	-	3.35 (.88)	-	-
Self-esteem	-	4.54 (1.73)	-	-
Hopelessness	-	2.48 (.97)	-	-
Locus of control (power)	-	2.83 (.88)	-	-
Locus of control (chance)	-	2.76 (.74)	-	-
Locus of control (internal)	-	3.65 (.69)	-	-
**Social capital and belonging**				
Practical support	4084 (94.8)	-	3657 (90.2)	397 (9.8)
People to socialise with	4064 (94.4)	-	3642 (90.2)	395 (9.8)

For peer review only

Neighbourhood belonging	3518 (82.1)	-	3176 (90.8)	322 (9.21)

For peer review only

Supplementary File Table 3. *Logistic regression predicting suicidal ideation excluding (Model 1) and including (Model 2) mental health variables.*

	Model 1			Model 2	
Predictors	*Adjusted Odds Ratio of suicidal ideation*	*95% CI*		*Adjusted Odds Ratio of suicidal ideation*	*95% CI*
Mental health					
Depression	-	-		*7.24****	*5.22, 10.07*
Anxiety	-	-		*1.56***	*1.13, 2.17*
Paranoia	-	-		*1.36**	*1.72*
Wellbeing	-	-		*.76*	*.56, 1.01*
Demographics					
Age (65+)					
18-24	*5.50****	*2.74, 11.06*		*0.95*	*.38, 2.38*
25-44	*4.50****	*2.48, 8.15*		*1.62*	*.84, 3.15*
45-64	*2.82****	*1.68, 4.73*		*1.1*	*.60, 2.02*
Female	*.88*	*.67, 1.17*		*.81*	*.57, 1.14*
Black & Minority Ethnic (BME)	*1.88**	*1.01, 3.49*		*1.93**	*1.04, 3.62*
LGBTQ	*1.93*	*.77, 4.83*		*2.73**	*1.00, 7.46*
Single/never married or civil partnership	*1.16*	*.87, 1.56*		*1.07*	*.74, 1.56*

For peer review only

Socioeconomic status					
Education (No qualifications)					
Professional, vocational or work Certificate	*.97*	*.71, 1.34*		*.76*	*.52, 1.12*
Degree or higher	*1.06*	*.67, 1.67*		*.70*	*.40, 1.21*
Non-employment	*1.43**	*1.00, 2.03*		*1.06*	*.68, 1.65*
Problems with housing	*1.67****	*1.26, 2.23*		*1.34*	*.95, 1.89*
Financial position (worse)					
Same	*1.68**	*1.02, 2.76*		*2.29***	*1.24, 4.23*
Better	*1.59*	*.91, 2.77*		*1.19*	*.58, 2.42*
Caring responsibilities					
None					
1-19 hours/week	*0.69*	*.38, 1.24*		*.64*	*.32, 1.30*
20-49 hours/week	*1.24*	*.61, 2.53*		*1.00*	*.39, 2.57*
50+ hours/week	*1.23*	*.65, 2.33*		*.68*	*.31, 1.49*
Health problems (EQ-5D)					
Pain	*1.62**	*1.09, 2.40*		*0.98*	*.61, 1.56*
Self-care	*1.38*	*.83, 2.27*		*1.02*	*.50, 2.08*
Usual activities	*1.02*	*.64, 1.62*		*0.64*	*.35, 1.18*
Mobility	*1.00*	*.64, 1.55*		*1.12*	*.65, 1.93*
Health conditions					
Cancer	*1.74*	*.80, 3.77*		*3.90***	*1.40, 10.84*
Diabetes	*1.03*	*.58, 1.83*		*.86*	*.43, 1.75*
Epilepsy	*1.73*	*.98, 3.06*		*1.65*	*.80, 3.39*

For peer review only

Migraine	*1.27*	*.83, 1.94*		*0.73*	*.39, 1.35*
Dementia	*.71*	*.26, 1.98*		*0.29*	*.06, 1.47*
Eye	*.97*	*.64, 1.48*		*1.23*	*.74, 2.06*
Ear	*2.02***	*1.20, 3.41*		*1.24*	*.59, 2.59*
Stroke	*2.01**	*1.06, 3.81*		*1.63*	*.57, 4.68*
Heart	*.80*	*.47, 1.37*		*1.3*	*.68, 2.48*
Blood pressure	*1.19*	*.47, 1.80*		*1.3*	*.77, 2.18*
Bronchitis/Emphysema	*1.28*	*.65, 2.51*		*2*	*.84, 4.80*
Asthma	*1.11*	*.69, 1.76*		*0.95*	*.51, 1.77*
Allergies	*1.09*	*.56, 2.13*		*0.89*	*.31, 2.53*
Stomach/digestive	*1.06*	*.63, 1.77*		*1.18*	*.59, 2.37*
Liver	*1.06*	*.53, 2.12*		*0.68*	*.29, 1.58*
Bowel/colon	*1.54*	*.90, 2.62*		*1.64*	*.81, 3.32*
Bladder	*1.35*	*.73, 2.51*		*0.76*	*.35, 1.64*
Arthritis	*.59**	*.40, .88*		*.54**	*.30, .95*
Bone, back, joint, muscle	*.76*	*.52, 1.10*		*0.89*	*.53, 1.49*
Gout	*.46*	*.12, 1.83*		*0.57*	*.06, 5.40*
Skin	*.62*	*.35, 1.09*		*0.49*	*.23, 1.04*
Other	*.93*	*.57, 1.51*		*1.06*	*.60, 1.88*
Mental health comorbidity					
No conditions					
Mental health condition(s)	-	-		*1.46*	*.67, 3.16*
Physical health condition(s)	-	-		*0.78*	*.45, 1.36*
Physical & mental health condition(s)	-	-		*1.02*	*.49, 2.10*
Side effects					
No medication					
Never bothers	*1.47**	*1.02, 2.12*		*1.25*	*.77, 2.03*

For peer review only

Bothers a little	*2.93***	*1.35, 6.36*		*1.72*	*.64, 4.67*
Bothers somewhat	*2.31***	*1.23, 4.34*		*0.83*	*.37, 1.86*
Bothers a lot	*2.64***	*1.37, 5.10*		*0.72*	*.25, 1.93*

For peer review only

Alcohol consumption					
Moderate (<14 units)					
None (0 units)	*.63***	*.46, .87*		*.61**	*.42, .90*
Heavy (14-28 units)	*.77*	*.45, 1.34*		*0.53*	*.23, 1.21*
Very heavy (>28 units)	*.51*	*.25, 1.02*		*0.66*	*.31, 1.39*
Smoking status					
Never					
Past occasional smoking	*1.52*	*.88, 2.65*		*1.65*	*.82, 3.32*
Past daily smoking	*1.24*	*.81, 1.90*		*1.05*	*.62, 1.77*
Current occasional smoking	*1.99**	*1.04, 3.81*		*1.78*	*.80, 3.96*
Current daily smoking	*1.92****	*1.35, 2.74*		*1.51*	*.98, 2.33*
Psychological factors					
Empathy	*0.82**	*.70, .96*		*.72***	*.59, .88*
Self-esteem	*.81****	*.75, .88*		*.97*	*.87, 1.09*
Hopelessness	*1.20**	*1.02, 1.41*		*.93*	*.75, 1.15*
Locus of control (power)	*1.08*	*.90, 1.30*		*.89*	*.71, 1.11*
Locus of control (chance)	*1.35***	*1.11, 1.64*		*1.23*	*.95, 1.60*
Locus of control (internal)	*.95*	*.76, 1.19*		*1.05*	*.82, 1.36*
Social capital					
Practical support	*.70*	*.30, 1.61*		*.67*	*.26, 1.70*
Socialise	*1.28*	*.63, 2.61*		*1.57*	*.63, 3.92*
Neighbourhood Belonging	*.69**	*.48, .97*		*.90*	*.58, 1.38*

* $p < .05$, ** $p < .01$, *** $p < .001$

For peer review only

Reporting checklist for cross sectional study.

Based on the STROBE cross sectional guidelines.

Instructions to authors

Complete this checklist by entering the page numbers from your manuscript where readers will find each of the items listed below.

Your article may not currently address all the items on the checklist. Please modify your text to include the missing information. If you are certain that an item does not apply, please write "n/a" and provide a short explanation.

Upload your completed checklist as an extra file when you submit to a journal.

In your methods section, say that you used the STROBE cross sectionalreporting guidelines, and cite them as:

von Elm E, Altman DG, Egger M, Pocock SJ, Gotzsche PC, Vandenbroucke JP. The Strengthening the Reporting of Observational Studies in Epidemiology (STROBE) Statement: guidelines for reporting observational studies.

		Reporting Item	Page Number
**Title and abstract**			
Title	#1a	Indicate the study's design with a commonly used term in the title or the abstract	1
Abstract	#1b	Provide in the abstract an informative and balanced summary of what was done and what was found	2

For peer review only

Introduction

Background / rationale	#2	Explain the scientific background and rationale for the investigation being reported	4
Objectives	#3	State specific objectives, including any prespecified hypotheses	7

Methods

Study design	#4	Present key elements of study design early in the paper	7
Setting	#5	Describe the setting, locations, and relevant dates, including periods of recruitment, exposure, follow-up, and data collection	7-8
Eligibility criteria	#6a	Give the eligibility criteria, and the sources and methods of selection of participants.	7-8
	#7	Clearly define all outcomes, exposures, predictors, potential confounders, and effect modifiers. Give diagnostic criteria, if applicable	7-8
Data sources / measurement	#8	For each variable of interest give sources of data and details of methods of assessment (measurement). Describe comparability of assessment methods if there is more than one group. Give information separately for for exposed and unexposed groups if applicable.	9-10 & Supplementary File

For peer review only

Bias	#9	Describe any efforts to address potential sources of bias	10-11
Study size	#10	Explain how the study size was arrived at	7-8
Quantitative variables	#11	Explain how quantitative variables were handled in the analyses. If applicable, describe which groupings were chosen, and why	9-10 & Supplementary File
Statistical methods	#12a	Describe all statistical methods, including those used to control for confounding	10-11
Statistical methods	#12b	Describe any methods used to examine subgroups and interactions	10-11
Statistical methods	#12c	Explain how missing data were addressed	11
Statistical methods	#12d	If applicable, describe analytical methods taking account of sampling strategy	10-11
Statistical methods	#12e	Describe any sensitivity analyses	10-11
**Results**			
Participants	#13a	Report numbers of individuals at each stage of study—eg numbers potentially eligible, examined for eligibility, confirmed eligible, included in the study, completing follow-up, and analysed. Give information	10-11

For peer review only

		separately for for exposed and unexposed groups if applicable.	
Participants	#13b	Give reasons for non-participation at each stage	10-11
Participants	#13c	Consider use of a flow diagram	
Descriptive data	#14a	Give characteristics of study participants (eg demographic, clinical, social) and information on exposures and potential confounders. Give information separately for exposed and unexposed groups if applicable.	Supplementary File
Descriptive data	#14b	Indicate number of participants with missing data for each variable of interest	Supplementary file
Outcome data	#15	Report numbers of outcome events or summary measures. Give information separately for exposed and unexposed groups if applicable.	12-16
Main results	#16a	Give unadjusted estimates and, if applicable, confounder-adjusted estimates and their precision (eg, 95% confidence interval). Make clear which confounders were adjusted for and why they were included	Supplementary File
Main results	#16b	Report category boundaries when continuous variables were categorized	na
Main results	#16c	If relevant, consider translating estimates of relative risk into absolute risk for a meaningful time period	na

For peer review only

Other analyses	#17	Report other analyses done—e.g., analyses of subgroups and interactions, and sensitivity analyses	11
**Discussion**			
Key results	#18	Summarise key results with reference to study objectives	17
Limitations	#19	Discuss limitations of the study, taking into account sources of potential bias or imprecision. Discuss both direction and magnitude of any potential bias.	19-20
Interpretation	#20	Give a cautious overall interpretation considering objectives, limitations, multiplicity of analyses, results from similar studies, and other relevant evidence.	17-19
Generalisability	#21	Discuss the generalisability (external validity) of the study results	17; 19-21
**Other Information**			
Funding	#22	Give the source of funding and the role of the funders for the present study and, if applicable, for the original study on which the present article is based	22

None The STROBE checklist is distributed under the terms of the Creative Commons Attribution License CC-BY. This checklist can be completed online using https://www.goodreports.org/, a tool made by the EQUATOR Network in collaboration with Penelope.ai

For peer review only
